# Responses of African Savanna Trees to Large Herbivore Extinction and Rewilding

**DOI:** 10.1111/ele.70360

**Published:** 2026-03-12

**Authors:** Tyler C. Coverdale, Mahesh Sankaran, Andrew B. Davies, Jayashree Ratnam, Benjamin J. Wigley, David J. Augustine

**Affiliations:** ^1^ Department of Biological Sciences University of Notre Dame Notre Dame Indiana USA; ^2^ National Centre for Biological Sciences, TIFR Bangalore India; ^3^ Department of Organismic and Evolutionary Biology Harvard University Cambridge Massachusetts USA; ^4^ Savanna Node, Scientific Services, SANParks Skukuza South Africa; ^5^ School of Natural Resource Management Nelson Mandela University George South Africa; ^6^ Plant Ecology University of Bayreuth Bayreuth Germany; ^7^ USDA‐ARS Rangeland Resources and Systems Research Unit Fort Collins Colorado USA

**Keywords:** African savanna, demography, extinction, herbivore exclosure, light detection and ranging, megafauna, Mpala Research Centre, plant‐herbivore interactions, remote sensing, trophic rewilding

## Abstract

The global decline or extinction of large mammals over the last 50,000 years has caused sweeping changes in the ecosystems they once inhabited. Trophic rewilding holds promise for returning lost ecological function and restoring processes that support ecosystem resilience, but there remains considerable uncertainty surrounding the efficacy of rewilding. To address this uncertainty, we experimentally excluded a diverse African savanna mammal community from replicated plots for 18 years to simulate extinction. Herbivore exclusion caused a rapid increase in tree cover, which was underlain by shifts in community composition and increases in canopy area, growth rate and density. We then removed the exclosure fences, simulating rewilding. Reintroducing herbivores rapidly reduced tree cover and largely reversed individual phenotypic shifts, but tree density remained elevated despite increased mortality rates after reintroduction. Our results suggest that even short‐term extirpation can cause complex shifts in vegetation communities, some of which may be resistant to rewilding.

## Graphical abstract

Trophic rewilding may be a restoration ‘win‐win’ if the return of extirpated wildlife also restores lost ecosystem function, but few studies have addressed whether wildlife reintroduction is capable of reversing changes that occurred during extirpation. We experimentally excluded a diverse African savanna large herbivore community for 18 years, which caused changes at individual, population and community levels that ultimately increased tree cover. After reintroducing wildlife into formerly fenced plots, many, but not all, of these changes were reversed, suggesting that some impacts of extirpation may be resistant to rewilding.

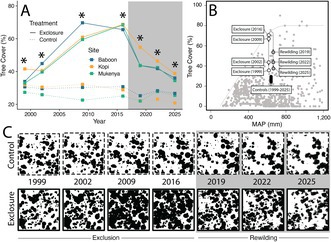

## 1 Introduction

Between 50,000 and 10,000 years ago, a combination of human activity and climate variability drove 40%–60% of terrestrial large mammal genera extinct throughout much of North and South America, Europe and Australia (Gill 2014; Owen‐Smith 1989). Human‐driven extinctions have continued in a size‐biased fashion to the present day, resulting in the extinction of more than 300 large mammal species in the last 500 years alone; even among species that have persisted through this period, average population sizes have declined by nearly 25% (Dirzo et al. 2014). Although the timing and intensity of these more recent declines vary across ecosystems, few large mammal communities remain intact (Galetti et al. 2018). Among large mammals, herbivores currently face the highest extinction threat (Atwood et al. 2020) and, by virtue of their trophic position, their absence has had cascading effects on the structure, function and service provision of plant communities worldwide (Pringle et al. 2023).

Against this backdrop of growing extinction threat (Cowie et al. 2022), the possibility of regaining lost ecological functions through trophic rewilding—the reintroduction of extirpated wildlife or functionally similar proxies—has become the subject of lively debate (see, for example, Bakker and Svenning 2018; Hart et al. 2023; O'Connell and Prudhomme 2024; Perino et al. 2019; Svenning et al. 2016; Torres et al. 2018). Past rewilding efforts, which include several widely known examples (e.g., the reintroduction of wolves into Yellowstone National Park) and many more opportunistic reintroductions (e.g., of elephants into the conservancies surrounding Kruger National Park), have collectively demonstrated that reintroducing extinct megafauna can have sweeping direct and indirect effects on ecosystem structure and function (Svenning et al. 2016). However, only a minority of rewilding efforts have been accompanied by the baseline data and post‐reintroduction monitoring necessary to mechanistically understand the ecological impacts of wildlife reintroduction. As a result, robust evidence for the efficacy of rewilding is currently lacking and, despite its intuitive appeal as a tool for regaining lost ecological function, many ecologists have argued that calls to implement trophic rewilding are not supported by empirical evidence (Gordon et al. 2023; Hart et al. 2023; O'Connell and Prudhomme 2024; Perino et al. 2019; Svenning et al. 2016).

African savannas largely escaped the Late Quaternary extinctions. Despite recent human‐driven declines (Ripple et al. 2015), many African savannas still support near‐historic diversities and densities of large mammalian herbivores (Stuart 2015). Savanna herbivores—including African elephants ( *Loxodonta africana* ; Laws 1970), white rhinoceros ( *Ceratotherium simum* ; Waldram et al. 2008), hippopotamus ( *Hippopotamus amphibius* ; Voysey et al. 2023), impala ( *Aepyceros melampus* ; Ford et al. 2014) and others (Coetsee et al. 2023)—have been widely studied, and insights gained from their experimental exclusion have provided critical mechanistic insights into the functioning of savanna ecosystems and the consequences of ongoing herbivore declines (Ripple et al. 2015; Sankaran et al. 2013). Given the presumed ecological similarities between extant African megafauna and extinct megafauna elsewhere in the world, these same exclosure studies have also proven useful for identifying the global consequences of Quaternary megafauna extinctions (Bakker et al. 2016; Bakker and Svenning 2018).

While excluding herbivores through fencing can shed light on the effects of herbivore extinction, reintroducing herbivores into long‐term exclosure studies can help address the uncertainties surrounding rewilding. Although such planned reintroductions are rare, they provide significant advantages over unplanned or incidental rewilding ‘experiments’ (Svenning et al. 2016; Tanentzap and Smith 2018), chief among which are that responses to reintroduction can be contextualised within an a priori framework of causal biotic interactions informed by the effects of exclusion (Gordon et al. 2023). Deliberate reintroduction following experimental exclusion also addresses the widespread but untested assumption—challenged by the ubiquity of ecological context‐dependence (*sensu* Catford et al. 2022)—that rewilding can completely reverse the ecological impacts of extinction. Understanding these potentially opposing effects is particularly critical in the context of herbivore extinction, which has well‐documented direct and indirect effects on ecosystem function mediated by impacts on vegetation (Pringle et al. 2023; Ripple et al. 2015).

Here, we used experimental large‐herbivore exclosures to investigate the responses of a Kenyan savanna tree community to 18 years of herbivore exclusion (simulating extinction) and 8 years of herbivore reintroduction (simulating rewilding). Across both phases of the experiment, we quantified the effects of large mammals on vegetation through the lens of tree cover, which is a major driver of ecosystem dynamics in savannas (Staver et al. 2011) and emerges from the collective effects of browsers on individual tree morphology, growth, survival and recruitment, as well as population density and community composition. The impacts of savanna browsers of diverse body size—including elephant, giraffe ( *Giraffa camelopardalis* ), impala and dik‐dik (*Madoqua* cf. *guentheri*)—on these individual‐, population‐ and community‐scale responses are well documented (e.g., Abraham et al. 2021; Davies and Asner 2019; Gordon et al. 2023; Kimuyu et al. 2021; Laws 1970), including during the first 10 years of our exclosure study (Sankaran et al. 2013). Accordingly, we hypothesized that experimental herbivore exclusion would cause tree cover to increase to a rainfall‐imposed maximum (Sankaran et al. 2005) and that this increase would be driven by predictable, directional responses at individual and population scales (e.g., increased recruitment, canopy area, density and cover). We further hypothesized that these same changes would cause the reintroduction of herbivores to have more idiosyncratic effects on the tree community due to the context‐dependence of tree‐herbivore interactions. In particular, changes in tree cover and density following reintroduction might proceed more slowly if increased height and canopy area caused by exclusion result in ‘escape’ (*sensu* Bond et al. 2012) from top‐down control, or may proceed more quickly if relaxation of antiherbivore defences during the period of exclusion makes trees more vulnerable to browsing (Wigley et al. 2019). Given the comparatively short height required for trees to become insensitive to mesobrowsers at our site (Augustine and McNaughton 2004), we hypothesized that changes in tree cover and density would proceed more slowly in response to reintroduction than to exclusion.

## 2 Materials and Methods

### 2.1 Study Site and Herbivore Exclosure Experiment

All research was conducted at Mpala Research Centre (MRC), which encompasses ~20,000 ha of semi‐arid, thorn‐scrub savanna in Laikipia County, Kenya. The study site is underlain by infertile red sandy loams, which support a tree community dominated by spinescent *Acacia s.l*. (including *Senegalia* and *Vachellia* spp.) species, notably *A. etbaica* and *A. mellifera* , as well as *Grewia tenax*. Another spinescent acacia, *A. brevispica*, is also abundant but was not included as a focal species because it did not occur within one of our exclosure sites. Approximately 40 other trees species (e.g., *Balanites rotundifolia*, *Croton dichogamus* ) occur at lower densities (Alston et al. 2022; Coverdale et al. 2024; Mutuku and Kenfack 2019). The understory consists of several hundred species of grasses, forbs and subshrubs, which form a discontinuous herbaceous layer (Alston et al. 2022; Augustine 2003; Coverdale et al. 2016). Mean annual rainfall (2000–2015) at the site averages 540–640 mm year^−1^, and occurs primarily during three discrete wet seasons (April–May, August and October; Augustine and McNaughton 1998, 2004); rainfall is typically higher in the southern region of Mpala, though there is pronounced interannual variation in the timing and amount of precipitation across the site (Augustine and McNaughton 2006).

More than 20 species of native large (≥ 5 kg) mammalian herbivores occur at MRC (Appendix S1), which falls within the high variety and abundance of larger species herbivome (‘high VALS’ *sensu* Hempson et al. 2015). Among non‐domesticated browsers, elephant (~3000 kg km^−2^), impala (~800 kg km^−2^), dik‐dik (~700 kg km^−2^) and giraffe ( *G. camelopardalis reticulata* , ~250 kg km^−2^) achieve the highest biomass densities (Augustine 2010). These species have non‐redundant effects on the tree community at MRC, which collectively impose strong top‐down control on tree demography (Sankaran et al. 2013). Elephant (and, to a lesser extent, giraffe) are capable of shaping individual tree canopies and can be a major source of tree mortality across size classes (Coverdale et al. 2016; Davies and Asner 2019); accordingly, we expect the exclusion and reintroduction of elephants to have strong effects on individual tree growth and mortality, total density and cover. Among medium‐bodied browsers, impala are the most abundant species and, in addition to browsing on seedlings, exert strong top‐down control on the growth of 0.5–1.5 m tall trees at our study site (Sankaran et al. 2013); accordingly, we expect impala (and other similarly sized browsers and mixed‐feeders) to primarily control the growth of intermediate sized trees and seedling recruitment. Dik‐dik, which are the most numerically abundant large herbivore and the dominant small‐bodied browser at MRC, impose a ‘browse trap’ on saplings < 0.5 m tall and consume seedlings in the understory (Sankaran et al. 2013); accordingly, we expect dik‐dik to exert strong top‐down control on recruitment and sapling growth.

In 1999, three pairs of 0.5 ha (70 × 70 m) large‐herbivore exclosure plots and unfenced controls were constructed, with two (Kopi and Baboon) in the southern (0.295137° N, 36.883456° E and 0.303034° N, 36.888642° E) and one (Mukenya) in the central (0.379699° N, 36.879494° E) regions of MRC. Although the sites encompass a slight rainfall gradient (Sankaran et al. 2013), the abundance and identity of browsers and mixed feeders are consistent across sites (Goheen et al. 2013). At each site, the exclosure plot consisted of a 3‐m tall, 11‐strand electrified fence that excluded all large herbivores ≥ 5 kg. Paired control plots were marked with wooden posts. Additional details concerning site characteristics and exclosure design can be found in Augustine and McNaughton (2004, 2006). Exclosure fences were maintained—with occasional, short‐term incursions by large herbivores (primarily dik‐dik)—until 2017, when they were fully removed. Thus, the experiment comprised two phases: an initial exclusion phase from 1999 to 2017, during which all large mammalian herbivores were excluded and a subsequent reintroduction phase from 2017 to 2025, when all large mammalian herbivores were reintroduced.

To quantify the effects of browser exclusion and reintroduction on the tree community, we performed five field surveys during which we mapped and measured every tree ≥ 50 cm tall within a central 50 × 50 m grid in each exclosure and control plot (Augustine and McNaughton 2004). Each individual tree was marked with a unique numbered tag to facilitate repeat measurements over time. Comprehensive field surveys were repeated in 1999, 2002, 2009, 2016 and 2019. The first survey was conducted in May–June 1999, which coincided with the construction of the fences and thus provided pre‐manipulation baseline data. The next three surveys occurred during the period of herbivore exclusion (1999–2017), with the 2016 surveys occurring the year before fences were removed. The 2019 survey, which was the last comprehensive field survey, occurred approximately 2 years after the reintroduction of large herbivores into exclosure plots. To quantify changes in canopy cover beyond the 2019 field survey, we also collected high‐resolution light detection and ranging (LiDAR) data from all experimental plots in January 2022 and February 2025, which extended our record of tree cover responses to 8 years after fence removal. Our LiDAR data acquisition and processing pipeline is described in detail in Appendix S2.

For all field surveys, we recorded the species, location (*x* and *y* coordinate within the central grid), canopy diameters (in both E‐W and N‐S cardinal directions) and maximum height of all trees. From these data, we estimated 10 aspects of tree responses to experimental herbivore exclusion and reintroduction that span scales of organisation from individuals to communities. At the individual scale, we compared (i) tree height and (ii) canopy area, which were measured directly in the field. We also quantified (iii) height growth and (iv) canopy area growth by dividing the change in height and canopy area, respectively, by the number of years between surveys for each tree. For canopy area growth, we also analysed data separately for each of the three dominant species and all other species pooled together. At the population scale, we quantified (v) tree density by counting the total number of individual trees ≥ 50 cm in each plot. Changes in tree density reflected the counterbalancing processes of (vi) recruitment and (vii) mortality, which we quantified by summing the number of new trees ≥ 50 cm in each plot and the number of trees that died during each inter‐survey period, respectively. Finally, at the community scale, we quantified composition by calculating (viii) the proportion and (ix) total tree canopy area of *A. etbaica*, *A. mellifera* and *G. tenax* and all other species pooled together. Finally, we estimated (*x*) canopy cover by aligning our elliptical estimates of individual canopy area with the *x* and *y* coordinates of each stem; the resulting plot maps provide estimates of aerial cover by accounting for canopy overlap, which is substantial at our site.

To evaluate the effects of experimental herbivore exclusion and reintroduction (i.e., fence removal) over time, we compared all responses using linear mixed models with treatment (exclosure vs. control) as a fixed effect, site as a random effect to account for the randomised complete block design and year as a random effect with plot as the subject to account for the repeated measures component of the study design. Tree height and canopy area (and their respective rates of change over time), density, recruitment, mortality and canopy cover (total and by species category) were averaged at the plot level (*N* = 3 plots/treatment) prior to analysis. Because recruitment, mortality and growth rates were calculated over time, these analyses contained only four time points (corresponding to the end of each growth period: 2002, 2009, 2016 and 2019). Analyses of tree height, canopy area, density, cover and community composition included 1999 as an additional time point. Analyses of tree cover included all five field surveys and two additional LiDAR surveys in 2022 and 2025. For the analyses of growth and total canopy area by species category, we used species category (*A. etbaica*, *A. mellifera* , *G. tenax* and all other species) nested within plot as the subject that was measured repeatedly over time. For the analysis of total canopy area by species, we square‐root transformed cover values to meet model assumptions. For each response variable, we fit models that assumed the covariance matrix either followed a compound symmetry, unstructured or a first‐order autoregressive structure and selected the covariance structure that both met the assumption of normality of residuals and minimised Akaike's Information Criterion. In cases where we found a significant Treatment × Year × Species interaction, we then evaluated contrasts between treatment and control for each Year × Species combination. In a few cases where the model residuals displayed heteroscedasticity, we square‐root transformed the response variable, which homogenised the residuals. We consider significant differences between exclosure treatments after 1999 (when fences were installed) and prior to 2017 (when fences were removed) to be evidence of tree responses to simulated herbivore extinction. Likewise, we consider the attenuation of exclosure treatment differences (or the appearance of novel differences) after 2017 to be evidence of tree responses to herbivore reintroduction. Full model outputs can be found in Appendix S3.

## 3 Results

### 3.1 Individual‐Scale Responses

**FIGURE 1:**
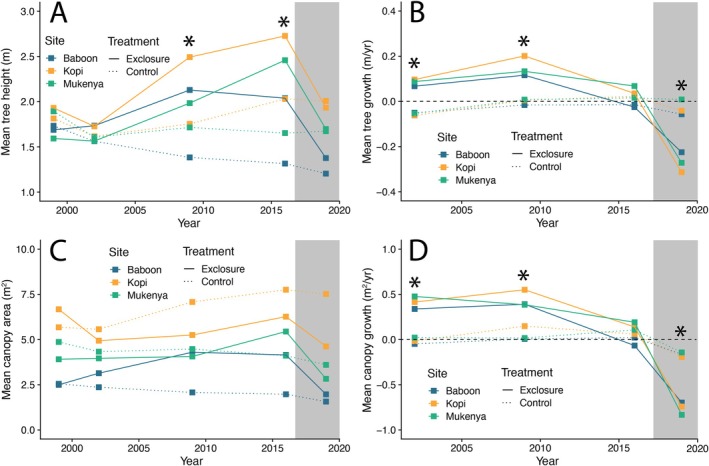
Changes in individual tree morphology in response to herbivore exclosure (2002–2016) and rewilding (2019); surveys in 1999 represent pre‐exclosure baselines. (A) Mean tree height. (B) Mean tree height growth. (C) Mean canopy area. (D) Mean canopy area growth. Data are from field surveys of individual trees and are averaged at the plot level (*N* = 3 plots/treatment; individual plots denoted with different colours). Growth rates are measured by calculating the change in canopy area between sequential survey years and dividing by the number of years between surveys; rates are reported for the end date of each intersurvey period (e.g., rates displayed in 2002 represent annual growth rates between 1999 and 2002). Solid lines denote exclosure plots; dashed lines denote unfenced control plots. The dashed horizontal line in (B and D) denotes zero average growth (i.e., no change in height or canopy area over time). The grey box denotes the period of herbivore reintroduction, which occurred 1 year after the 2016 surveys. (*) denotes significant treatment differences at *p <* 0.05.

**FIGURE 2:**
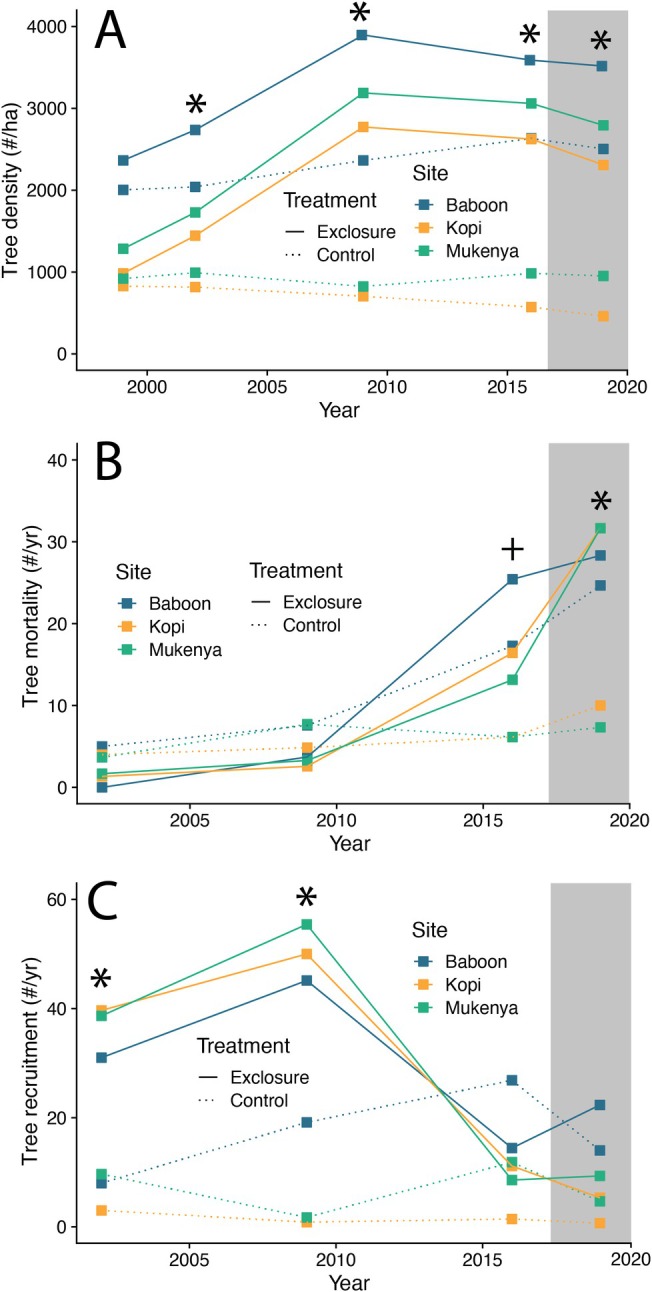
Changes in tree population dynamics in response to herbivore exclusion (2002–2016) and rewilding (2019); surveys in 1999 represent pre‐exclosure baselines. (A) Tree density. (B) Tree mortality. (C) Tree recruitment. Data are from field surveys of individual trees and are averaged at the plot level (*N* = 3 plots/treatment; individual plots denoted with different colours). Mortality and recruitment rates are reported for the end year of each time period (e.g., rates displayed in 2002 represent mortality or recruitment rates between 1999 and 2002). Solid lines denote exclosure plots; dashed lines denote unfenced control plots. The grey box denotes the period of herbivore reintroduction, which occurred 1 year after the 2016 surveys. (*) denotes significant treatment differences at *p <* 0.05; (+) denotes significant treatment differences at 0.05 < *p <* 0.10.

Mean tree height did not differ between exclosure and control plots for the first 4 years of the experiment (1999–2003; Figure 1A), but during every phase of the experiment, trees in unfenced control plots exposed to browsers experienced negative or neutral height growth, on average (Figure 1B). In contrast, trees in exclosure plots experienced positive height growth from 1999 to 2009 (Figure 1B; Treatment effect: *F*_*1*,*2*_ = 17.4, *p* = 0.053), which slowed to levels comparable to control plots from 2009 to 2016. Although individual growth rates in the absence of herbivores were positive, the average height of trees in two of the three exclosure plots decreased between 1999 and 2002 due to increased recruitment of new trees (Figure 2C), which had a mean height (± SE) of 77.7 ± 1.5 cm across all exclosure plots in 2002. Consistent with the difference in growth rates across treatments during the period of herbivore exclusion, trees in exclosure plots were nearly 1 m taller (mean height: 2.37 m) than trees in unfenced control plots (mean height: 1.49 m) by 2016 (17 years after the initiation of exclosures). During the first 3 years following fence removal (2016–2019), trees in formerly fenced plots experienced the greatest negative height growth observed across all plots in any phase of the experiment (Treatment × Year effect: *F*_*3*,*12*_ = 43.97, *p* < 0.0001), resulting in no difference in mean tree height across treatments only 2 years after herbivore reintroduction (Treatment × Year effect: *F*_*4*,*16*_ = 6.61, *p* = 0.0024).

Mean canopy area did not differ between exclosure and control plots at any point during the 18 years of herbivore exclusion or the 2 years of herbivore reintroduction (Figure 1C; Treatment × Year effect: *F*_*1*,*14.1*_ = 1.3, *p* = 0.32). However, despite no differences in mean canopy area, canopy area growth was significantly greater in exclosure plots than control plots for the first 10 years of exclusion (Figure 1D); similar to changes in tree height over time, we suggest that this combination of results is consistent with the greater recruitment of smaller individuals into exclosure plots having a dampening effect on mean canopy area but not growth. Between 2009 and 2016 canopy area growth was similar across treatments. During the first 2 years of herbivore reintroduction, trees in newly unfenced plots experienced negative canopy growth, on average (Treatment × Year effect: *F*_*3*,*7.11*_ = 309.5, *p* < 0.0001). The responses of two of the three dominant tree species (and all other species lumped as a single category) mirrored this pattern: *Grewia tenax* and *Acacia mellifera* tended to experience positive canopy growth in exclosure plots during the exclosure phase and neutral or negative growth in control plots across all phases. Both species experienced negative canopy area growth after herbivore reintroduction. In contrast, *A. etbaica* was the only species to experience positive canopy growth in the presence of herbivores and had significantly greater growth in exclosures during only one period (2009–2016) of the exclosure phase (Figure S1). The size distribution of all trees was similar across exclosure treatments at the onset of the experiment (Figure S2). Between 2009 and 2016, a greater proportion of trees in exclosure plots had larger canopies, but after 2 years of herbivore reintroduction this difference had essentially disappeared.

### 3.2 Population‐Scale Responses

At the onset of the experiment, tree density did not vary between exclosure and control plots, though there was a nearly 2.5‐fold difference in tree density across sites (Figure 2A). Between 1999 (mean density in exclosures: 1544 trees ha^−1^) and 2009 (mean density in exclosures: 3285 trees ha^−1^), tree density doubled in exclosures but remained essentially unchanged across the three control plots (Treatment effect: *F*_*1*,*3.03*_ = 54.23, *p* = 0.005). In pairwise comparisons within year, tree density was significantly (*p* < 0.05) greater in exclosure plots than control plots from 2002 to 2019, which included both the exclusion and reintroduction phases of the experiment (Figure 2A). After 2016, tree density in the exclosure plots remained relatively static (decreasing by only *c*. 12.5%) through 2019, 2 years after fences were removed (Treatment × Year effect: *F*_*4*,*15.9*_ = 15.9, *p* < 0.0001).

Tree mortality was low (< 10 trees years^−1^) and indistinguishable across treatments between 1999 and 2009 (Figure 2B), after which mortality rates nearly quadrupled in exclosure plots (mean mortality in exclosures between 2009 and 2016: 19.2 trees years^−1^). Mortality rates increased further in each exclosure plot following the removal of exclosure fences, ultimately reaching an average mortality rate of 30.5 trees years^−1^; for comparison, average mortality rates in the control plots between 2016 and 2019 were 14.0 trees years^−1^, and with the exception of one site (Baboon) did not vary appreciably across years (Treatment × Year effect: *F*_*3*,*9.7*_ = 5.05, *p* = 0.02). Tree recruitment was greater in exclosure plots than control plots during the first 10 years of exclusion (Figure 2C). In the 7 years prior to fence removal (2009–2016), recruitment in exclosure plots decreased by nearly 70% and remained low for 2 years following fence removal; recruitment did not vary appreciably across years in the control plots (Treatment × Year effect: *F*_*3*,*12*_ = 18.2, *p* < 0.0001).

### 3.3 Community‐Scale Responses

**FIGURE 3:**
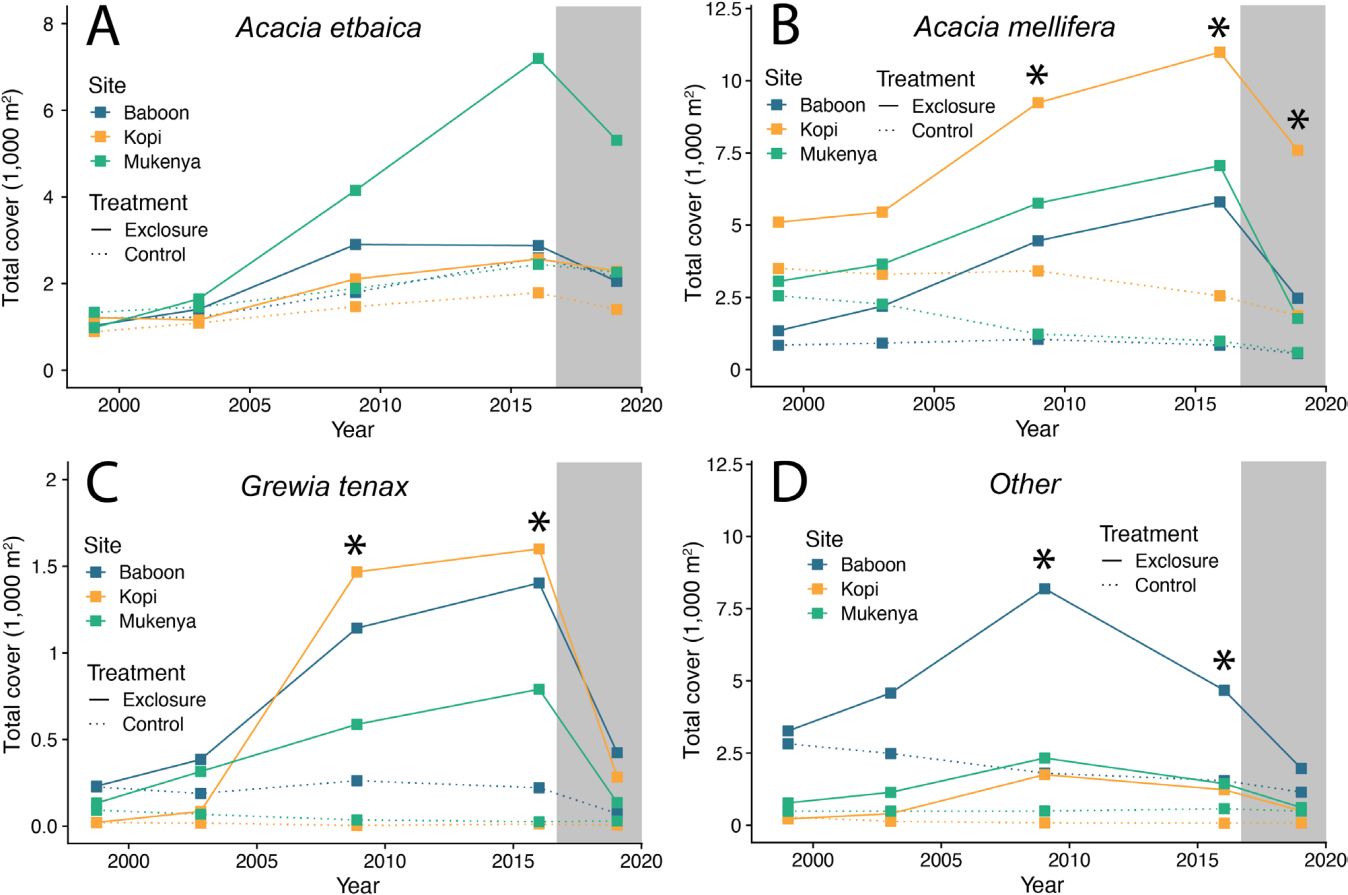
Changes in tree cover by species in response to herbivore exclusion (2002–2016) and rewilding (2019); surveys in 1999 represent pre‐exclosure baselines. (A) *Acacia etbaica*. (B) *A. mellifera* . (C) *Grewia tenax*. (D) All other species. Data are from field surveys of individual trees and are averaged at the plot level (*N* = 3 plots/treatment; individual plots denoted with different colours). Note that *y*‐axis scales differ between panels. Solid lines denote exclosure plots; dashed lines denote unfenced control plots. The grey box denotes the period of herbivore reintroduction, which occurred 1 year after the 2016 surveys. (*) denotes significant treatment differences at *p <* 0.05. See also Figure S3, which represents a density‐based estimate of community composition.

There was a significant Species × Treatment × Year effect for our density‐based estimate of community composition (*F*_*12*,*18.9*_ = 2.92, *p* = 0.02), but no species differed in proportional abundance across treatments at any point in the experiment (all pairwise *F*_*1*,*15‐17*_ < 0.23, *p* > 0.64; Figure S3). In contrast, the absolute cover of the three dominant tree species did vary across years and treatments when measured in terms of total canopy area (Treatment × Species × Year effect: *F*_*12*,*64*_ = 1.92, *p* = 0.048; Figure 3). In particular, *A. etbaica* was the only species to increase in total cover in the unfenced control plots, where it nearly doubled from an average of 1152 m^2^ per plot in 1999 to a high of 2284 m^2^ per plot in 2016 (Figure 3A). Every other species decreased in total cover in unfenced control plots over both phases of the experiment (Figure 3B–D). During the exclosure phase (1999–2016), *A. etbaica*, *A. mellifera* and all other species also approximately doubled within exclosure plots; in contrast, *G. tenax* increased ~8‐fold in exclosure plots between 1999 and 2016. Of the three dominant species, only *A. mellifera* remained elevated relative to controls following reintroduction, though this difference was driven largely by a single site (Kopi) with very high *A. mellifera* cover (Figure 3B).

**FIGURE 4:**
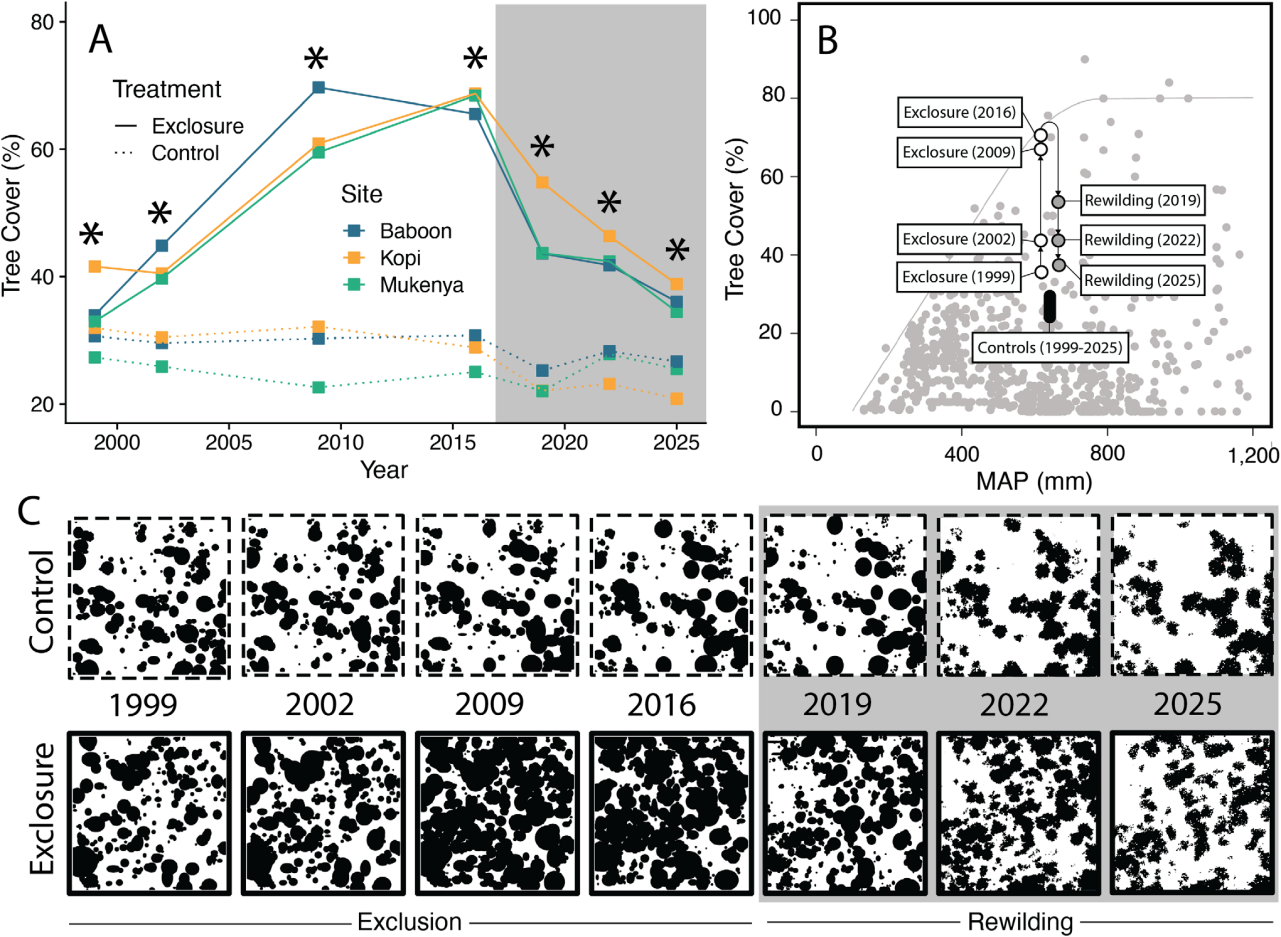
Change in tree cover over time in response to herbivore exclosure (2002–2016) and rewilding (2019–2025); surveys in 1999 represent pre‐exclosure baselines. (A) Change in tree cover. (*) denotes significant treatment differences at *p <* 0.05. (B) Variation in tree cover over time in relation to the rainfall‐imposed upper limit; underlying figure modified from Sankaran et al. (2005). Average rainfall at MRC is 540–640 mm years^−1^. Points are jittered for clarity and do not reflect interannual variation in rainfall. (C) Representative changes in tree cover (black) for a single paired exclosure and control plot (Baboon). The grey box denotes the period of rewilding, which began in 2017. Cover data from 1999 to 2019 are from field surveys; cover data from 2022 and 2025 are from LiDAR surveys.

Tree cover was stable across years in the three unfenced control plots, where it ranged from 21% to 32% over time (Figure 4A). In contrast, tree cover increased rapidly during the first 10 years of herbivore exclusion and, by 2009, exceeded 60% cover, on average (Treatment effect: *F*_*1*,*4.86*_ = 234.3, *p* < 0.0001). This level of cover remained stable through 2016 (Figure 4A,B). In pairwise comparisons across treatments within years, tree cover was greater in exclosure plots in every year from 1999 (when it was ~7% greater, on average) to 2016 (when it was ~40% greater). Following the reintroduction of herbivores in 2017, tree cover declined at a comparable rate to the increase during the first 10 years of exclusion: by 2025, tree cover in formerly fenced plots was nearly identical to initial cover values (Figure 4C; Treatment × Year effect: *F*_*6*,*20.3*_ = 16.5, *p* < 0.0001), but remained slightly elevated relative to control plots.

## 4 Discussion

Our study took advantage of a rare opportunity to reintroduce an intact large‐herbivore community to sites that had been experimentally fenced for nearly two decades. By simulating total extinction and full rewilding, we were able to evaluate existing theories about the structure of African savannas (e.g., rainfall limitation of tree cover; Sankaran et al. 2005), and more generally examine the realised effects of trophic rewilding. Below, we discuss the implications of our results through the lens of tree cover.

### 4.1 Tree Responses to Herbivore Exclusion

Disturbance, including browsing by large mammalian herbivores, is one of the major determinants of tree cover in African savannas and is theorised to explain variation in tree cover below the maximum threshold imposed by rainfall (Sankaran et al. 2005). The results of our study support this notion. Our study site receives ~540–640 mm of rain annually, which situates it at the upper end of the rainfall‐limited range of tree cover in African savannas. At the onset of the experiment, tree cover in our fenced plots averaged ~40%, which was well below the theorised maximum cover (~80%) at this site (Figure 4B). In the absence of herbivores, tree cover increased to a steady maximum of ~75% within 10 years (see also Sankaran et al. 2013), and remained at this level until after we reintroduced herbivores 7 years later. Comparable increases in woody cover in the absence of herbivores have been noted in savannas throughout Africa (Daskin et al. 2016; Holdo et al. 2009; Stevens et al. 2017), underscoring the important role of large herbivores in regulating vegetation physiognomy.

The rapid increase in tree cover we observed could have been caused by any of several non‐mutually exclusive mechanisms, including (i) a shift in tree community composition toward larger canopied or faster growing species, (ii) an increase in individual tree size and/or (iii) an increase in tree density through increased recruitment and/or decreased mortality. Our results suggest that all three mechanisms likely contributed to the doubling of tree cover during the first 10 years of the experiment. Although the proportional density of the dominant species did not vary over time or across treatments (Figure S3), the total canopy cover of all species increased in the absence of herbivores (Figure 3), a result consistent with previous research in our exclosure plots (Sankaran et al. 2013). The increase in canopy cover in exclosure plots was particularly pronounced for *A. mellifera* (Figure 3B), which also exhibited the greatest individual canopy growth rate in the absence of browsers (Figure S1). Individual tree size also responded strongly to herbivore exclusion and was a major contributor to increased tree cover. Although individual trees within exclosure plots experienced greater canopy growth than those in unfenced controls (Figure 1D, Figure S1), average individual canopy area at the plot level did not vary across treatments during the period of exclusion. These apparently conflicting results are consistent with the significantly greater recruitment of juvenile trees in exclosure plots immediately following exclusion (Figure 2C), which increased tree density (Figure 2A) and contributed to positive canopy growth (Figure 1D) while depressing average canopy size (Figure 1C). Although it would not have contributed to our measures of tree cover, individual tree height also increased significantly in exclosure plots, consistent with the greater annual vertical growth of trees during the period of exclusion (Figure 1A,B) and potential escape from the ‘browse trap’ (Staver and Bond 2014). Of note, all these changes occurred within the first 3 years of exclusion, a result we expect is at least partially due to the complete exclusion of herbivores from our plots: in contrast to true extinctions, which are typically preceded by decades or centuries of size‐biased population declines (Atwood et al. 2020; Gill 2014; Owen‐Smith 1989), experimental exclosures typically simulate a complete and instantaneous extinction event.

Although we measured a broad suite of traits that encompassed individual‐, population‐ and community‐scale responses to herbivore exclusion, other changes may have occurred during the exclusion phase that our measures did not capture but that are nonetheless relevant to the observed responses to herbivore reintroduction. Previous research in this experiment (Wigley et al. 2019) suggests that 10 years of herbivore exclusion is sufficient to relax physical defence investment and alter chemical defence traits in dominant *Acacia* species. Induction of physical and chemical defences by herbivores following reintroduction may reduce subsequent risk to individual trees, but previous studies at MRC suggest that defence induction in ‘naïve’ *Acacia* is variable across individuals and may take more than a year to occur (Gadd et al. 2001; Young et al. 2003). Increasing tree density may also contribute to a more resource‐acquisitive phenotype, ultimately resulting in more ‘pole‐like’ canopy architectures consistent with greater tree heights in exclosure plots (Figure 1A; Staver and Bond 2014) that are more vulnerable to herbivores than the ‘cage‐like’ architecture typically adopted by dominant tree species at our site (Ford et al. 2014; Wigley et al. 2019). These and other phenotypic shifts occurring in the absence of herbivores may make individual trees more susceptible to subsequent browsing (Young et al. 2021).

In addition to individual phenotypic shifts, increased tree density and cover in the exclosure plots contributed to stronger tree‐tree competition (Dohn et al. 2017), potentially resulting in non‐linear demographic and compositional responses to herbivore exclusion. For example, *A. etbaica* initially experienced greater growth in the absence of herbivores than in their presence, but this trend reversed between 2009 and 2016, prior to the reintroduction of herbivores (Figure S1B). This shift is not likely the result of increased recruitment of juvenile *A. etbaica* during this time, as overall recruitment in the exclosure plots dropped by more than 50% (Figure 2C) and mortality increased two‐fold (Figure 2B) between 2009 and 2016. Instead, this result is consistent with greater individual stress and self‐thinning caused by intensifying tree‐tree competition in low disturbance environments (Sea and Hanan 2012), specifically in the absence of large herbivores (Dohn et al. 2017). Because fire is almost completely suppressed at our study site, it is also likely that intensifying tree‐grass competition caused by the absence of herbivores further suppressed recruitment and tree growth particularly for saplings and smaller trees. Notably, *A. etbaica* is the most physically defended of the dominant trees at MRC and was the only species to experience increased total canopy cover in the presence of herbivores (Figure 3A). Collectively, these results suggest that co‐existence among dominant tree species at our site may be facilitated by spatial or temporal refugia from browsers, which may allow for the persistence of more poorly defended species; conversely, in the absence of browsers, *A. etbaica* may eventually be outcompeted by species that invest less in antiherbivore defenses.

### 4.2 Tree Responses to Herbivore Reintroduction

The decline of tree cover following herbivore reintroduction was nearly identical to the increase in tree cover following herbivore exclusion: in both cases, cover changed rapidly during the first decade before stabilising either at the rainfall‐imposed maximum (by 2009) or at pre‐exclosure levels (by 2025; Figure 4A,B). Although tree cover remained elevated in reintroduction plots (compared to controls) in 2025, it had returned to levels nearly identical to those observed at the onset of the experiment (1999 exclosure average cover: 36.2%; 2025 reintroduction average cover: 36.5%). Many of the responses we measured followed a similarly symmetrical pattern of shifting directionally in response to herbivore exclusion and reverting at comparable rates after reintroduction. Tree height (Figure 1A) and recruitment (Figure 2C, Figure S2), for example, quickly returned to pre‐exclosure levels following the reintroduction of herbivores, consistent with prolonged, concentrated browsing on adult trees by elephants following fence removal and recolonization of plots by major seedling and sapling browsers (e.g., dik‐dik and impala), respectively (Augustine and McNaughton 2004; Sankaran et al. 2013). We suggest that the rapidity of these reversals may be partially due to the comparatively short period of exclusion followed by instantaneous and complete reintroduction of large herbivores in our study, but we also note that similarly rapid reversals of woody encroachment have been noted in other systems, many of which experienced more prolonged periods without herbivores before rewilding or experienced only partial reintroduction of large mammal communities (e.g., Garrido et al. 2021; Gordon et al. 2023; Guyton et al. 2020; Young et al. 2021).

Other aspects of the tree community responded more individualistically (*sensu* Gleason 1926) during the exclusion and reintroduction phases. For example, nearly all species experienced roughly zero net height growth in control plots for the duration of the experiment (Figure S1), suggesting that, for most species, vertical growth is entirely offset by losses to large mammalian browsers. The only species to exhibit positive individual (height; Figure S1B) and population (total cover; Figure 3A) growth in control plots was *A. etbaica*, which similarly increased in basal area in the presence of browsers during the first 10 years of our experiment (Sankaran et al. 2013). In exclosure plots, in contrast, nearly every species exhibited positive height growth due to the loss of top‐down control from large browsers. However, the average height of all species (Figure S1) declined following herbivore reintroduction, a result consistent with a specific type of ecological context‐dependence that may be widespread in rewilding contexts: individual phenotypic shifts and changing interspecific interactions in the absence of herbivores may result in vegetation communities more susceptible to herbivores following their reintroduction (Young et al. 2021). In the specific case of *Acacia* trees in African savannas, the induction of defences following prolonged herbivore exclusion requires damage to poorly defended trees (which itself limits growth), is variable across individuals, and may take more than a year to occur (Gadd et al. 2001; Young et al. 2003). As a result, large mammalian browsers may be able to exert stronger top‐down control on tree growth over the first several years following reintroduction than on trees constantly exposed to herbivory (Young et al. 2021).

In the context of the broader rewilding debate, variables that respond to simulated extinction but fail to revert following rewilding are of particular interest, as they suggest that the prolonged absence of herbivores can cause recalcitrant shifts in the outcome of species interactions (i.e., ecological context dependence). In our study, tree density was the most notable property that followed this pattern: tree density was elevated in exclosure plots within 3 years, continued to increase during the exclosure phase, and remained high 3 years after reintroduction. We suggest that this pattern is consistent with an escape from top‐down control during the exclosure phase. In particular, the increased density of trees in fenced plots appears to have been driven primarily by increased growth (Figure 1D) of juvenile trees, ultimately resulting in a prolonged period of elevated recruitment (Figure 2C). Whereas browsing almost completely offset tree growth in control plots during this time (Figure 1B, Figure 3) and suppressed the recruitment of new individuals (Figure 2C), trees in exclosure plots grew ~10 cm years^−1^ for the first 10 years of the study and ~5 cm years^−1^ between 2009 and 2016. As a result, the smallest trees that we included in our initial surveys in 1999 were, on average, nearly 1.8 m tall by the time browsers were reintroduced. Critically, this height is above the maximum browsing height of both dik‐dik and impala (DuToit 1990) and is within the height class (1.5–2.5 m) in which mesobrowsers do not appreciably affect tree growth at our study site (Augustine and McNaughton 2004). Furthermore, trees in this intermediate height class experience much less bark‐stripping damage from elephants compared to trees taller than 2.5 m (Augustine and McNaughton 2004). Thus, it appears that 18 years of simulated extinction allowed many trees to escape the mesobrowser trap, which more than offset the elevated mortality following herbivore reintroduction (Figure 2B) and ultimately resulted in a higher density tree community with comparable overall canopy cover (Figure 4).

Although our study is among the first to reintroduce large mammalian herbivores following a prolonged period of experimental exclusion, several important caveats bear mentioning. First, fire is a major determinant of tree cover across African savannas (Holdo et al. 2009; Staver et al. 2011), but is almost entirely absent from our study site. Whether trees in exclosure plots would have escaped the browse trap and achieved persistently elevated densities with fire present is unknown, but we consider it likely that elevated herbaceous fuel loads caused by total herbivore exclusion would have increased the frequency and intensity of fires, potentially slowing tree recruitment and sapling growth. Second, although the duration of our exclusion study was long by the standards of ecological experiments, compared to recent extinction events (e.g., the Pleistocene extinctions) it was short, instantaneous and complete. Fencing designs that exclude subsets of intact large herbivore communities (e.g., Goheen et al. 2013) and more gradual reintroductions following exclusion would more realistically mimic current rewilding efforts. Finally, the rewilding phase of our experiment was also comparatively short, and we consider it likely that ongoing successional changes (facilitated by herbivore disturbance) will continue to shape the tree community in our reintroduction plots. Whether the changes we observed are transient or permanent will ultimately inform our understanding of successional dynamics in savannas, which are poorly understood relative to other ecosystems (Van Langevelde et al. 2003).

## 5 Conclusion

Quantifying the strength, direction and scale of large herbivore impacts on vegetation communities is critical not only for understanding the functioning of present‐day ecosystems, but also for contextualising the impacts of past extinctions and informing future rewilding efforts. Past studies have used large herbivore exclosures to unravel these interactions, and here we show that reintroducing wildlife can reveal changes caused by short‐term exclusion that are recalcitrant to rewilding. Additional studies that contextualise rewilding within an a priori mechanistic framework will be useful for determining how common these changes are across ecosystems, with the important caveat that the duration, rapidity and completeness of experimental exclusion and reintroduction often differ from true extinction and rewilding events.

## Author Contributions

Tyler C. Coverdale and David J. Augustine conceived the research plan. Mahesh Sankaran, Jayashree Ratnam, Benjamin J. Wigley and David J. Augustine conceived and coordinated the exclosure experiment. Tyler C. Coverdale and Andrew B. Davies collected and processed remote sensing data. Tyler C. Coverdale and David J. Augustine analysed field data. Tyler C. Coverdale analysed remote sensing data and wrote the manuscript. All authors contributed to and approved the final manuscript.

## Funding

This work was supported by the Harvard University Star‐Friedman Challenge for Promising Scientific Research. National Centre for Biological Sciences. The University of Notre Dame. Natural Environment Research Council, NE‐E017436‐1. National Geographic Society, #982815.

## Supporting information


**Data S1:** ele70360‐sup‐0001‐Appendices.docx.

## Data Availability

Data and code supporting the results of this manuscript have been deposited on Figshare (DOI: 10.6084/m9.figshare.30076576).

## References

[ele70360-bib-0001] Abraham, J. O. , E. R. Goldberg , J. Botha , and A. C. Staver . 2021. “Heterogeneity in African Savanna Elephant Distributions and Their Impacts on Trees in Kruger National Park, South Africa.” Ecology and Evolution 11: 5624–5634. 34026034 10.1002/ece3.7465 PMC8131780

[ele70360-bib-0002] Alston, J. M. , C. G. Reed , L. M. Khasoha , et al. 2022. “Ecological Consequences of Large Herbivore Exclusion in an African Savanna: 12 Years of Data From the UHURU Experiment.” Ecology 103: e3649. 35084743 10.1002/ecy.3649

[ele70360-bib-0003] Atwood, T. B. , S. A. Valentine , E. Hammill , et al. 2020. “Herbivores at the Highest Risk of Extinction Among Mammals, Birds, and Reptiles.” Science Advances 6: eabb8458. 32923612 10.1126/sciadv.abb8458 PMC7457337

[ele70360-bib-0004] Augustine, D. J. 2003. “Spatial Heterogeneity in the Herbaceous Layer of a Semi‐Arid Savanna Ecosystem.” Plant Ecology 167: 319–332.

[ele70360-bib-0005] Augustine, D. J. 2010. “Response of Native Ungulates to Drought in Semi‐Arid Kenyan Rangeland.” African Journal of Ecology 48: 1009–1020.

[ele70360-bib-0006] Augustine, D. J. , and S. J. McNaughton . 1998. “Ungulate Effects on the Functional Species Composition of Plant Communities: Herbivore Selectivity and Plant Tolerance.” Journal of Wildlife Management 62: 1165–1183.

[ele70360-bib-0007] Augustine, D. J. , and S. J. McNaughton . 2004. “Regulation of Shrub Dynamics by Native Browsing Ungulates on East African Rangeland.” Journal of Applied Ecology 41: 45–58.

[ele70360-bib-0008] Augustine, D. J. , and S. J. McNaughton . 2006. “Interactive Effects of Ungulate Herbivores, Soil Fertility, and Variable Rainfall on Ecosystem Processes in a Semi‐Arid Savanna.” Ecosystems 9: 1242–1256.

[ele70360-bib-0009] Bakker, E. S. , J. L. Gill , C. N. Johnson , et al. 2016. “Combining Paleo‐Data and Modern Exclosure Experiments to Assess the Impact of Megafauna Extinctions on Woody Vegetation.” Proceedings of the National Academy of Sciences 113: 847–855. 10.1073/pnas.1502545112 PMC4743795 26504223

[ele70360-bib-0010] Bakker, E. S. , and J. C. Svenning . 2018. “Trophic Rewilding: Impact on Ecosystems Under Global Change.” Philosophical Transactions of the Royal Society, B: Biological Sciences 373: 20170432. 10.1098/rstb.2017.0432 PMC6231072 30348876

[ele70360-bib-0011] Bond, W. , G. Cook , and R. Williams . 2012. “Which Trees Dominate in Savannas? The Escape Hypothesis and Eucalypts in Northern Australia.” Austral Ecology 37: 678–685.

[ele70360-bib-0012] Catford, J. A. , J. R. U. Wilson , P. Pyšek , P. E. Hulme , and R. P. Duncan . 2022. “Addressing Context Dependence in Ecology.” Trends in Ecology & Evolution 37: 158–170. 34756764 10.1016/j.tree.2021.09.007

[ele70360-bib-0013] Coetsee, C. , J. Botha , M. F. Case , A. Manganyi , and F. Siebert . 2023. “The Hard Lives of Trees in African Savanna—Even Without Elephants.” Austral Ecology 48: 532–551.

[ele70360-bib-0014] Coverdale, T. C. , P. B. Boucher , J. Singh , et al. 2024. “Herbivore Regulation of Savanna Vegetation: Structural Complexity, Diversity, and the Complexity–Diversity Relationship.” Ecological Monographs 94: e1624.

[ele70360-bib-0015] Coverdale, T. C. , T. Kartzinel , K. Grabowski , et al. 2016. “Elephants in the Understory: Opposing Direct and Indirect Effects of Consumption and Ecosystem Engineering by Megaherbivores.” Ecology 97: 3219–3230. 27870025 10.1002/ecy.1557

[ele70360-bib-0016] Cowie, R. H. , P. Bouchet , and B. Fontaine . 2022. “The Sixth Mass Extinction: Fact, Fiction or Speculation?” Biological Reviews 97: 640–663. 35014169 10.1111/brv.12816 PMC9786292

[ele70360-bib-0017] Daskin, J. H. , M. Stalmans , and R. M. Pringle . 2016. “Ecological Legacies of Civil War: 35‐Year Increase in Savanna Tree Cover Following Wholesale Large‐Mammal Declines.” Journal of Ecology 104: 79–89.

[ele70360-bib-0018] Davies, A. B. , and G. P. Asner . 2019. “Elephants Limit Aboveground Carbon Gains in African Savannas.” Global Change Biology 25: 1368–1382. 30723962 10.1111/gcb.14585

[ele70360-bib-0019] Dirzo, R. , H. S. Young , M. Galetti , G. Ceballos , N. J. B. Isaac , and B. Collen . 2014. “Defaunation in the Anthropocene.” Science 345: 401–406. 25061202 10.1126/science.1251817

[ele70360-bib-0020] Dohn, J. , D. J. Augustine , N. P. Hanan , J. Ratnam , and M. Sankaran . 2017. “Spatial Vegetation Patterns and Neighborhood Competition Among Woody Plants in an East African Savanna.” Ecology 98: 478–488. 27864944 10.1002/ecy.1659

[ele70360-bib-0021] DuToit, J. 1990. “Feeding‐Height Stratification Among African Browsing Ruminants.” African Journal of Ecology 28: 55–61.

[ele70360-bib-0022] Ford, A. T. , J. R. Goheen , T. O. Otieno , et al. 2014. “Large Carnivores Make Savanna Tree Communities Less Thorny.” Science (1979) 346: 346–349. 10.1126/science.1252753 25324387

[ele70360-bib-0023] Gadd, M. E. , T. P. Young , and T. M. Palmer . 2001. “Effects of Simulated Shoot and Leaf Herbivory on Vegetative Growth and Plant Defense in *Acacia drepanolobium* .” Oikos 92: 515–521.

[ele70360-bib-0024] Galetti, M. , M. Moleón , P. Jordano , et al. 2018. “Ecological and Evolutionary Legacy of Megafauna Extinctions.” Biological Reviews 93: 845–862. 28990321 10.1111/brv.12374

[ele70360-bib-0025] Garrido, P. , L. Edenius , G. Mikusiński , A. Skarin , A. Jansson , and C.‐G. Thulin . 2021. “Experimental Rewilding May Restore Abandoned Wood‐Pastures if Policy Allows.” Ambio 50: 101–112. 32152907 10.1007/s13280-020-01320-0 PMC7708577

[ele70360-bib-0026] Gill, J. L. 2014. “Ecological Impacts of the Late Quaternary Megaherbivore Extinctions.” New Phytologist 201: 1163–1169. 24649488 10.1111/nph.12576

[ele70360-bib-0027] Gleason, H. A. 1926. “The Individualistic Concept of the Plant Association.” Bulletin of the Torrey Botanical Club 53: 7–26.

[ele70360-bib-0028] Goheen, J. R. , T. M. Palmer , G. K. Charles , et al. 2013. “Piecewise Disassembly of a Large‐Herbivore Community Across a Rainfall Gradient: The UHURU Experiment.” PLoS One 8: e55192. 23405122 10.1371/journal.pone.0055192 PMC3566220

[ele70360-bib-0029] Gordon, C. E. , M. Greve , M. Henley , A. Bedetti , P. Allin , and J. Svenning . 2023. “Elephant Rewilding Affects Landscape Openness and Fauna Habitat Across a 92‐Year Period.” Ecological Applications 33: e2810. 36694991 10.1002/eap.2810

[ele70360-bib-0030] Guyton, J. A. , J. Pansu , M. C. Hutchinson , et al. 2020. “Trophic Rewilding Revives Biotic Resistance to Shrub Invasion.” Nature Ecology & Evolution 4: 712–724. 31932702 10.1038/s41559-019-1068-y

[ele70360-bib-0031] Hart, E. E. , A. Haigh , and S. Ciuti . 2023. “A Scoping Review of the Scientific Evidence Base for Rewilding in Europe.” Biological Conservation 285: 110243.

[ele70360-bib-0032] Hempson, G. P. , S. Archibald , and W. J. Bond . 2015. “A Continent‐Wide Assessment of the Form and Intensity of Large Mammal Herbivory in Africa.” Science 350: 1056–1061. 26612946 10.1126/science.aac7978

[ele70360-bib-0033] Holdo, R. M. , R. D. Holt , and J. M. Fryxell . 2009. “Grazers, Browsers, and Fire Influence the Extent and Spatial Pattern of Tree Cover in the Serengeti.” Ecological Applications 19: 95–109. 19323175 10.1890/07-1954.1

[ele70360-bib-0034] Kimuyu, D. M. , D. Kenfack , P. M. Musili , and R. O. Ang'ila . 2021. “Fine‐Scale Habitat Heterogeneity Influences Browsing Damage by Elephant and Giraffe.” Biotropica 53: 86–96.

[ele70360-bib-0035] Laws, R. M. 1970. “Elephants as Agents of Habitat and Landscape Change in East Africa.” Oikos 21: 1–15.

[ele70360-bib-0036] Mutuku, P. M. , and D. Kenfack . 2019. “Effect of Local Topographic Heterogeneity on Tree Species Assembly in an Acacia‐Dominated African Savanna.” Journal of Tropical Ecology 35: 46–53.

[ele70360-bib-0037] O'Connell, M. J. , and C. T. Prudhomme . 2024. “The Need for an Evidence‐Led Approach to Rewilding.” Journal for Nature Conservation 79: 126609.

[ele70360-bib-0038] Owen‐Smith, N. 1989. “Megafaunal Extinctions: The Conservation Message From 11,000 Years B.P.” Conservation Biology 3: 405–412. 21129027 10.1111/j.1523-1739.1989.tb00246.x

[ele70360-bib-0039] Perino, A. , H. M. Pereira , L. M. Navarro , et al. 2019. “Rewilding Complex Ecosystems.” Science 364: eaav5570. 31023897 10.1126/science.aav5570

[ele70360-bib-0040] Pringle, R. M. , J. O. Abraham , T. M. Anderson , et al. 2023. “Impacts of Large Herbivores on Terrestrial Ecosystems.” Current Biology 33: R584–R610. 37279691 10.1016/j.cub.2023.04.024

[ele70360-bib-0041] Ripple, W. J. , T. M. Newsome , C. Wolf , et al. 2015. “Collapse of the World's Largest Herbivores.” Science Advances 1: e1400103. 26601172 10.1126/sciadv.1400103 PMC4640652

[ele70360-bib-0042] Sankaran, M. , D. J. Augustine , and J. Ratnam . 2013. “Native Ungulates of Diverse Body Sizes Collectively Regulate Long‐Term Woody Plant Demography and Structure of a Semi‐Arid Savanna.” Journal of Ecology 101: 1389–1399.

[ele70360-bib-0043] Sankaran, M. , N. P. Hanan , R. J. Scholes , et al. 2005. “Determinants of Woody Cover in African Savannas.” Nature 438: 846–849. 16341012 10.1038/nature04070

[ele70360-bib-0044] Sea, W. B. , and N. P. Hanan . 2012. “Self‐Thinning and Tree Competition in Savannas.” Biotropica 44: 189–196.

[ele70360-bib-0045] Staver, A. C. , S. Archibald , and S. A. Levin . 2011. “Tree Cover in Sub‐Saharan Africa: Rainfall and Constrain Forest and Savanna as Alternative Stable States.” Ecology 92: 1063–1072. 21661567 10.1890/10-1684.1

[ele70360-bib-0046] Staver, A. C. , and W. J. Bond . 2014. “Is There a “Browse Trap”? Dynamics of Herbivore Impacts on Trees and Grasses in an African Savanna.” Journal of Ecology 102: 595–602.

[ele70360-bib-0047] Stevens, N. , C. E. R. Lehmann , B. P. Murphy , and G. Durigan . 2017. “Savanna Woody Encroachment Is Widespread Across Three Continents.” Global Change Biology 23: 235–244. 27371937 10.1111/gcb.13409

[ele70360-bib-0048] Stuart, A. J. 2015. “Late Quaternary Megafaunal Extinctions on the Continents: A Short Review.” Geological Journal 50: 338–363.

[ele70360-bib-0049] Svenning, J.‐C. , P. B. M. Pedersen , C. J. Donlan , et al. 2016. “Science for a Wilder Anthropocene: Synthesis and Future Directions for Trophic Rewilding Research.” Proceedings of the National Academy of Sciences 113: 898–906. 10.1073/pnas.1502556112 PMC4743824 26504218

[ele70360-bib-0050] Tanentzap, A. J. , and B. R. Smith . 2018. “Unintentional Rewilding: Lessons for Trophic Rewilding From Other Forms of Species Introductions.” Philosophical Transactions of the Royal Society, B: Biological Sciences 373: 20170445. 10.1098/rstb.2017.0445 PMC6231064 30348872

[ele70360-bib-0051] Torres, A. , N. Fernández , S. zu Ermgassen , et al. 2018. “Measuring Rewilding Progress.” Philosophical Transactions of the Royal Society, B: Biological Sciences 373: 20170433. 10.1098/rstb.2017.0433 PMC6231071 30348877

[ele70360-bib-0052] Van Langevelde, F. , C. A. D. M. Van De Vijver , L. Kumar , et al. 2003. “Effects of Fire and Herbivory on the Stability of Savanna Ecosystems.” Ecology 84: 337–350.

[ele70360-bib-0053] Voysey, M. D. , P. J. N. de Bruyn , and A. B. Davies . 2023. “Are Hippos Africa's Most Influential Megaherbivore? A Review of Ecosystem Engineering by the Semi‐Aquatic Common Hippopotamus.” Biological Reviews 98: 1509–1529. 37095627 10.1111/brv.12960

[ele70360-bib-0054] Waldram, M. S. , W. J. Bond , and W. D. Stock . 2008. “Ecological Engineering by a Mega‐Grazer: White Rhino Impacts on a South African Savanna.” Ecosystems 11: 101–112.

[ele70360-bib-0055] Wigley, B. J. , C. Coetsee , D. J. Augustine , J. Ratnam , D. Hattas , and M. Sankaran . 2019. “A Thorny Issue: Woody Plant Defence and Growth in an East African Savanna.” Journal of Ecology 107: 1839–1851.

[ele70360-bib-0056] Young, T. P. , D. M. Kimuyu , W. O. Odadi , H. B. M. Wells , and A. A. Wolf . 2021. “Naïve Plant Communities and Individuals May Initially Suffer in the Face of Reintroduced Megafauna: An Experimental Exploration of Rewilding From an African Savanna Rangeland.” PLoS One 16: e0248855. 33822786 10.1371/journal.pone.0248855 PMC8023473

[ele70360-bib-0057] Young, T. P. , M. L. Stanton , and C. E. Christian . 2003. “Effects of Natural and Simulated Herbivory on Spine Lengths of *Acacia drepanolobium* in Kenya.” Oikos 101: 171–179.

