# Supplementary material for: Responses of African Savanna Trees to Large Herbivore Extinction and Rewilding
Source: Ecol Lett. 2026 Mar 12;29(3):e70360. doi: 10.1111/ele.70360 (PMC12981617; doi:10.1111/ele.70360)
Supplement: Supplementary file 1 — Data S1: ele70360‐sup‐0001‐Appendices.docx. [file ELE-29-0-s001.docx]

**Supporting Information for:**

**Title:** Responses of African savanna trees to large herbivore extinction and rewilding

**Authors:** Tyler C. Coverdale^1^, Mahesh Sankaran^2^, Andrew B. Davies^3^, Jayashree Ratnam^2^, Benjamin J. Wigley^4,5,6^, and David J. Augustine^7^

**Author affiliations:** ^1^University of Notre Dame, Department of Biological Sciences, Indiana USA; ^2^Wildlife Biology and Conservation Program, National Centre for Biological Sciences, Bangalore, India; ^3^Harvard University, Department of Organismic and Evolutionary Biology, Massachusetts USA; ^4^Savanna Node, Scientific Services, SANParks, Skukuza, 1350, South Africa; ^5^School of Natural Resource Management, Nelson Mandela University, George Campus, George, 6530, South Africa; ^6^Plant Ecology, University of Bayreuth, Universitaetsstrasse 30, 95447, Bayreuth, Germany; ^7^USDA-ARS Rangeland Resources and Systems Research Unit, Colorado USA.

**Author emails:** mahesh@ncbs.res.in, andrew_davies@g.harvard.edu; j.ratnam.bangalore@gmail.com; benwigley@gmail.com; david.augustine@usda.gov

**Corresponding author information:** tcoverda@nd.edu; Ph: +1(574) 631-6552; Fax: +1(574) 631-7413

**This file includes:**

Appendix S1

Appendix S2

Supplementary Figures S1-S3

**Appendix S1:** List of large mammalian herbivores known to occur on the red soil at MRC

**Appendix S2:** Light detection and ranging (LiDAR) data collection and processing

**Appendix S3:** Statistical analyses

**Figure S1:** Annual growth of trees by exclosure treatment and species between 1999 and 2019

**Figure S2:** Distribution of canopy areas by exclosure treatment between 1999 and 2019

**Figure S3:** Density-based estimates of tree community composition between 1999 and 2019

**Supplementary methods**

**Appendix S1: List of large mammalian herbivores known to occur on the red soil at MRC**

The following native large mammalian herbivores are known to occur at Mpala Research Centre (adapted from Goheen et al. 2013).

- Buffalo (*Syncerus caffer*)
- Bush duiker (*Sylvicapra grimmia*)
- Bushbuck (*Tragelaphus sylvaticus*)
- Bushpig (*Potamochoerus larvatus*)
- Dik-dik (*Madoqua guentheri*)
- Eland (*Taurotragus oryx*)
- Elephant (*Loxodonta africana*)
- Gerenuk (*Litocranius walleri*)
- Grant's gazelle (*Nanger granti*)
- Greater kudu (*Tragelaphus strepsiceros*)
- Grevy's zebra (*Equus grevyi*)
- Hartebeest (*Alcelaphus buselaphus*)
- Hippopotamus (*Hippopotamus amphibius*)
- Impala (*Aepyceros melampus*)
- Klipspringer (*Oreotragus aureus*)
- Oryx (*Oryx beisa*)
- Plains zebra (*Equus quagga*)
- Reticulated giraffe (*Giraffa camelopardalis*)
- Steinbuck (*Raphicerus campestris*)
- Thomson's gazelle (*Eudorcas thomsoni*)
- Warthog (*Phacochoerus africanus*)
- Waterbuck (*Kobus defassa*)

**Appendix S2. Light detection and ranging (LiDAR) data collection and processing**

In this study, we collected high-resolution LiDAR data across all exclusion and control plots to extend our temporal record of tree cover. All flights were conducted with permission from the Kenya Civil Aviation Authority (2022: KCAA/OPS/2117/4; 2025: KCAA/OPS/2117/MPALA). In 2022, a Riegl VUX-1LR LiDAR sensor (integrated by Phoenix LiDAR Systems, Austin, Texas, USA) was flown using a Freefly Alta-X rotary-wing unoccupied aerial vehicle (UAV; Freefly Systems, Woodinville, Washington, USA). The UAV was flown at 8 m/s in a serpentine pattern at 50 m above ground with a line speed of 114 lines/second and an 820 kHz pulse rate (see Coverdale et al., 2024 for additional details). In 2025, a Riegl-U160 LiDAR sensor was flown using a Cessna 185. The plane was flown at an airspeed of 90-110 knots at ~240 m above ground with a line speed of 290 lines/second and a 600 kHz pulse rate. For both LiDAR surveys, flight trajectories were corrected during post-processing using GPS data from a nearby mobile base station. All LiDAR data were denoised, classified (Axelsson 2000), and aligned using the Terrasolid software suite (Terrasolid Ltd, Espoo, Finland). As a result of the different platforms, the denoised LiDAR datasets had different point densities (UAV: ~300 points m^-2^; plane: ~60 points m^-2^), but both were sufficient to quantify tree cover (Boucher et al., 2023). We created digital terrain models at 25-cm resolution for each site using a triangulated model of ground points. The height above ground was then computed for each point based on its vertical distance to the ground surface model, and the maximum height within each 25 cm x 25 cm pixel was used to create a rasterized canopy height model (CHM). Due to our inability to differentiate and identify individual trees with LiDAR, we were not able to directly compare all aspects of tree community composition, structure, and individual growth between LiDAR and field surveys. However, we were able to quantify tree cover, which we describe below.

Previous field surveys at MRC (Alston *et al.* 2022; Coverdale *et al.* 2024) suggest that more than 95% of herbaceous-layer vegetation is <1 m tall and more than 95% of the tree layer is >1 m tall; accordingly, we estimated tree cover in all plots by calculating the proportion of pixels in the CHM with points ≥1 m above ground level. This approach likely underestimates tree cover slightly by excluding trees <1 m tall (which were included in field surveys), but presently we are not able to directly differentiate tree and herbaceous-layer vegetation below 1 m with our remote sensing approach. For comparison, across all six exclosure and control plots in 1999 there were 657 trees shorter than 1 m. The total canopy area of these trees was 373.1 m^2^, which collectively represents ~2.5% of the tree cover per plot. The fact that many saplings are located beneath larger tree canopies (and therefore also do not contribute to field-derived estimates of tree cover due to canopy overlap) further bolsters our confidence that methodological differences in canopy cover estimates across years did not unduly compromise the rigor or robustness of our inferences. As a result, we included both LiDAR- and field-derived estimates of canopy cover in our analysis to investigate changes in tree cover over time in response to simulated herbivore extinction (1999-2016) and rewilding (2019-2025).

**Appendix S3: Statistical analyses**

In this study, we evaluated the effects of large mammalian herbivore exclusion and reintroduction on nine complementary response variables. Below, we include the full SAS model outputs for each response variable in the order they are presented in the Main Text:

**(i) Tree height**

| 1. **Model Information** | |
| --- | --- |
| **Data Set** | SASUSER.MPALAEXCL_HEIGHT |
| **Response Variable** | Height |
| **Response Distribution** | Gaussian |
| **Link Function** | Identity |
| **Variance Function** | Default |
| **Variance Matrix** | Not blocked |
| **Estimation Technique** | Restricted Maximum Likelihood |
| **Degrees of Freedom Method** | Kenward-Roger |
| **Fixed Effects SE Adjustment** | Kenward-Roger |

| **Class Level Information** | | |
| --- | --- | --- |
| **Class** | **Levels** | **Values** |
| **Treatment** | 2 | Control Exclosure |
| **Block** | 3 | Baboon Kopi Mukenya |
| **Subject** | 6 | 1 2 3 4 5 6 |
| **Year** | 5 | 1999 2002 2009 2016 2019 |

| **Number of Observations Read** | 30 |
| --- | --- |
| **Number of Observations Used** | 30 |

| **Dimensions** | |
| --- | --- |
| **G-side Cov. Parameters** | 3 |
| **R-side Cov. Parameters** | 1 |
| **Columns in X** | 18 |
| **Columns in Z** | 33 |
| **Subjects (Blocks in V)** | 1 |
| **Max Obs per Subject** | 30 |

| **Optimization Information** | |
| --- | --- |
| **Optimization Technique** | Dual Quasi-Newton |
| **Parameters in Optimization** | 3 |
| **Lower Boundaries** | 2 |
| **Upper Boundaries** | 0 |
| **Fixed Effects** | Profiled |
| **Residual Variance** | Profiled |
| **Starting From** | Data |

| **Iteration History** | | | | | |
| --- | --- | --- | --- | --- | --- |
| **Iteration** | **Restarts** | **Evaluations** | **Objective Function** | **Change** | **Max Gradient** |
| **0** | **0** | 4 | 10.385638415 | . | 59.67098 |
| **1** | **0** | 2 | 3.4566903509 | 6.92894806 | 1.830632 |
| **2** | **0** | 3 | 3.3298506985 | 0.12683965 | 1.761672 |
| **3** | **0** | 2 | 2.9548517337 | 0.37499896 | 1.551888 |
| **4** | **0** | 2 | 2.9382691399 | 0.01658259 | 1.726008 |
| **5** | **0** | 4 | 2.7209211075 | 0.21734803 | 0.437134 |
| **6** | **0** | 3 | 2.6845984254 | 0.03632268 | 0.178294 |
| **7** | **0** | 3 | 2.6736487044 | 0.01094972 | 0.044127 |
| **8** | **0** | 3 | 2.6735399783 | 0.00010873 | 0.011563 |
| **9** | **0** | 3 | 2.6735378809 | 0.00000210 | 0.000686 |
| **10** | **0** | 3 | 2.6735378688 | 0.00000001 | 2.026E-6 |

| Convergence criterion (GCONV=1E-8) satisfied. |
| --- |

| **Fit Statistics** | |
| --- | --- |
| **-2 Res Log Likelihood** | 2.67 |
| **AIC (smaller is better)** | 10.67 |
| **AICC (smaller is better)** | 13.34 |
| **BIC (smaller is better)** | 7.07 |
| **CAIC (smaller is better)** | 11.07 |
| **HQIC (smaller is better)** | 3.43 |
| **Generalized Chi-Square** | 0.57 |
| **Gener. Chi-Square / DF** | 0.03 |

| **Covariance Parameter Estimates** | | | |
| --- | --- | --- | --- |
| **Cov Parm** | **Subject** | **Estimate** | **Standard Error** |
| **Block** | Â | 0.03477 | 0.03780 |
| **Variance** | Subject | 0.001619 | 0.01064 |
| **CS** | Subject | -0.00018 | 0.006217 |
| **Residual** | Â | 0.02848 | . |

| **Solutions for Fixed Effects** | | | | | | | |
| --- | --- | --- | --- | --- | --- | --- | --- |
| **Effect** | **Treatment** | **Year** | **Estimate** | **Standard Error** | **DF** | **tÂ Value** | **Pr > \|t\|** |
| **Intercept** | Â | Â | 1.6688 | 0.1468 | 5.575 | 11.36 | <.0001 |
| **Year** | Â | 1999 | 0.06850 | 0.1417 | 16 | 0.48 | 0.6352 |
| **Year** | Â | 2002 | 0.006498 | 0.1417 | 16 | 0.05 | 0.9640 |
| **Year** | Â | 2009 | 0.5332 | 0.1417 | 16 | 3.76 | 0.0017 |
| **Year** | Â | 2016 | 0.7394 | 0.1417 | 16 | 5.22 | <.0001 |
| **Year** | Â | 2019 | 0 | . | . | . | . |
| **Treatment** | Control | Â | -0.04002 | 0.1412 | 16.8 | -0.28 | 0.7804 |
| **Treatment** | Exclosure | Â | 0 | . | . | . | . |
| **Treatment*Year** | Control | 1999 | 0.1163 | 0.2003 | 16 | 0.58 | 0.5696 |
| **Treatment*Year** | Control | 2002 | -0.03938 | 0.2003 | 16 | -0.20 | 0.8466 |
| **Treatment*Year** | Control | 2009 | -0.5444 | 0.2003 | 16 | -2.72 | 0.0152 |
| **Treatment*Year** | Control | 2016 | -0.7019 | 0.2003 | 16 | -3.50 | 0.0029 |
| **Treatment*Year** | Control | 2019 | 0 | . | . | . | . |
| **Treatment*Year** | Exclosure | 1999 | 0 | . | . | . | . |
| **Treatment*Year** | Exclosure | 2002 | 0 | . | . | . | . |
| **Treatment*Year** | Exclosure | 2009 | 0 | . | . | . | . |
| **Treatment*Year** | Exclosure | 2016 | 0 | . | . | . | . |
| **Treatment*Year** | Exclosure | 2019 | 0 | . | . | . | . |

| **Type III Tests of Fixed Effects** | | | | |
| --- | --- | --- | --- | --- |
| **Effect** | **Num DF** | **Den DF** | **F Value** | **PrÂ >Â F** |
| **Year** | 4 | 16 | 5.92 | 0.0040 |
| **Treatment** | 1 | 2 | 19.26 | 0.0482 |
| **Treatment*Year** | 4 | 16 | 6.61 | 0.0024 |

| **Treatment*Year Least Squares Means** | | | | | | |
| --- | --- | --- | --- | --- | --- | --- |
| **Treatment** | **Year** | **Estimate** | **Standard Error** | **DF** | **tÂ Value** | **Pr > \|t\|** |
| Control | 1999 | 1.8136 | 0.1468 | 5.575 | 12.35 | <.0001 |
| Control | 2002 | 1.5959 | 0.1468 | 5.575 | 10.87 | <.0001 |
| Control | 2009 | 1.6176 | 0.1468 | 5.575 | 11.02 | <.0001 |
| Control | 2016 | 1.6662 | 0.1468 | 5.575 | 11.35 | <.0001 |
| Control | 2019 | 1.6288 | 0.1468 | 5.575 | 11.09 | <.0001 |
| Exclosure | 1999 | 1.7373 | 0.1468 | 5.575 | 11.83 | <.0001 |
| Exclosure | 2002 | 1.6753 | 0.1468 | 5.575 | 11.41 | <.0001 |
| Exclosure | 2009 | 2.2019 | 0.1468 | 5.575 | 15.00 | <.0001 |
| Exclosure | 2016 | 2.4082 | 0.1468 | 5.575 | 16.40 | <.0001 |
| Exclosure | 2019 | 1.6688 | 0.1468 | 5.575 | 11.36 | <.0001 |

| **Tests of Effect Slices for Treatment*Year Sliced By Year** | | | | |
| --- | --- | --- | --- | --- |
| **Year** | **Num DF** | **Den DF** | **F Value** | **PrÂ >Â F** |
| 1999 | 1 | 16.8 | 0.29 | 0.5961 |
| 2002 | 1 | 16.8 | 0.32 | 0.5814 |
| 2009 | 1 | 16.8 | 17.12 | 0.0007 |
| 2016 | 1 | 16.8 | 27.60 | <.0001 |
| 2019 | 1 | 16.8 | 0.08 | 0.7804 |

**(ii) Canopy area**

| 1. **Model Information** | |
| --- | --- |
| **Data Set** | SASUSER.MPALAEXCL_AREA |
| **Response Variable** | Area |
| **Response Distribution** | Gaussian |
| **Link Function** | Identity |
| **Variance Function** | Default |
| **Variance Matrix** | Not blocked |
| **Estimation Technique** | Restricted Maximum Likelihood |
| **Degrees of Freedom Method** | Kenward-Roger |
| **Fixed Effects SE Adjustment** | Kenward-Roger |

| **Class Level Information** | | |
| --- | --- | --- |
| **Class** | **Levels** | **Values** |
| **Treatment** | 2 | Control Exclosure |
| **Block** | 3 | Baboon Kopi Mukenya |
| **Subject** | 6 | 1 2 3 4 5 6 |
| **Year** | 5 | 1999 2002 2009 2016 2019 |

| **Number of Observations Read** | 30 |
| --- | --- |
| **Number of Observations Used** | 30 |

| **Dimensions** | |
| --- | --- |
| **G-side Cov. Parameters** | 3 |
| **R-side Cov. Parameters** | 1 |
| **Columns in X** | 18 |
| **Columns in Z** | 33 |
| **Subjects (Blocks in V)** | 1 |
| **Max Obs per Subject** | 30 |

| **Optimization Information** | |
| --- | --- |
| **Optimization Technique** | Dual Quasi-Newton |
| **Parameters in Optimization** | 3 |
| **Lower Boundaries** | 3 |
| **Upper Boundaries** | 1 |
| **Fixed Effects** | Profiled |
| **Residual Variance** | Profiled |
| **Starting From** | Data |

| **Iteration History** | | | | | |
| --- | --- | --- | --- | --- | --- |
| **Iteration** | **Restarts** | **Evaluations** | **Objective Function** | **Change** | **Max Gradient** |
| **0** | **0** | 4 | 71.885503827 | . | 9.744213 |
| **1** | **0** | 4 | 67.010342915 | 4.87516091 | 17.44825 |
| **2** | **0** | 3 | 66.128789784 | 0.88155313 | 3.481465 |
| **3** | **0** | 2 | 65.060557828 | 1.06823196 | 6.442648 |
| **4** | **0** | 4 | 63.840011488 | 1.22054634 | 16.0375 |
| **5** | **0** | 2 | 63.100565882 | 0.73944561 | 2.331539 |
| **6** | **0** | 4 | 61.472414375 | 1.62815151 | 7.753589 |
| **7** | **0** | 3 | 60.971639449 | 0.50077493 | 5.464209 |
| **8** | **0** | 2 | 60.772079671 | 0.19955978 | 11.74024 |
| **9** | **0** | 2 | 60.44971367 | 0.32236600 | 3.919503 |
| **10** | **0** | 2 | 60.151423337 | 0.29829033 | 4.252687 |
| **11** | **0** | 3 | 59.956503757 | 0.19491958 | 0.742937 |
| **12** | **0** | 2 | 59.694552971 | 0.26195079 | 2.099008 |
| **13** | **0** | 3 | 59.605445466 | 0.08910751 | 2.647509 |
| **14** | **0** | 2 | 59.5426555 | 0.06278997 | 3.212237 |
| **15** | **0** | 2 | 59.500505957 | 0.04214954 | 1.859196 |
| **16** | **0** | 2 | 59.46810536 | 0.03240060 | 2.11519 |
| **17** | **0** | 2 | 59.444496181 | 0.02360918 | 1.440575 |
| **18** | **0** | 2 | 59.419615481 | 0.02488070 | 1.192597 |
| **19** | **0** | 2 | 59.398230371 | 0.02138511 | 1.424177 |
| **20** | **0** | 2 | 59.382655418 | 0.01557495 | 1.426174 |
| **21** | **0** | 2 | 59.373765829 | 0.00888959 | 1.156458 |
| **22** | **0** | 2 | 59.363979318 | 0.00978651 | 0.506383 |
| **23** | **0** | 2 | 59.355836651 | 0.00814267 | 1.000414 |
| **24** | **0** | 2 | 59.349358854 | 0.00647780 | 0.749685 |
| **25** | **0** | 3 | 59.345223992 | 0.00413486 | 0.236434 |
| **26** | **0** | 2 | 59.339422662 | 0.00580133 | 0.176482 |
| **27** | **0** | 2 | 59.336780276 | 0.00264239 | 0.777616 |
| **28** | **0** | 2 | 59.334297431 | 0.00248285 | 0.209423 |
| **29** | **0** | 3 | 59.333775546 | 0.00052188 | 0.150711 |
| **30** | **0** | 2 | 59.33293131 | 0.00084424 | 0.059553 |
| **31** | **0** | 2 | 59.331866512 | 0.00106480 | 0.003325 |
| **32** | **0** | 2 | 59.331194133 | 0.00067238 | 0.005453 |
| **33** | **0** | 2 | 59.331015666 | 0.00017847 | 0.040137 |
| **34** | **0** | 3 | 59.330960816 | 0.00005485 | 0.066702 |
| **35** | **0** | 2 | 59.330921897 | 0.00003892 | 0.072538 |
| **36** | **0** | 2 | 59.330897201 | 0.00002470 | 0.056331 |
| **37** | **0** | 2 | 59.330877304 | 0.00001990 | 0.040664 |
| **38** | **0** | 2 | 59.330865167 | 0.00001214 | 0.048908 |
| **39** | **0** | 2 | 59.330849475 | 0.00001569 | 0.008831 |
| **40** | **0** | 2 | 59.330826745 | 0.00002273 | 0.001361 |
| **41** | **0** | 2 | 59.330807248 | 0.00001950 | 0.01088 |
| **42** | **0** | 3 | 59.330805482 | 0.00000177 | 0.009237 |
| **43** | **0** | 2 | 59.330802634 | 0.00000285 | 0.002889 |
| **44** | **0** | 2 | 59.330799187 | 0.00000345 | 0.00008 |
| **45** | **0** | 2 | 59.330797365 | 0.00000182 | 0.001678 |

| Convergence criterion (GCONV=1E-8) satisfied. |
| --- |

| **Fit Statistics** | |
| --- | --- |
| **-2 Res Log Likelihood** | 59.33 |
| **AIC (smaller is better)** | 67.33 |
| **AICC (smaller is better)** | 70.00 |
| **BIC (smaller is better)** | 63.73 |
| **CAIC (smaller is better)** | 67.73 |
| **HQIC (smaller is better)** | 60.08 |
| **Generalized Chi-Square** | 0.00 |
| **Gener. Chi-Square / DF** | 0.00 |

| **Covariance Parameter Estimates** | | | |
| --- | --- | --- | --- |
| **Cov Parm** | **Subject** | **Estimate** | **Standard Error** |
| **Block** |  | 1.9776 | 1.7647 |
| **Variance** | Subject | 1.2563 | 1.2427 |
| **AR(1)** | Subject | 0.8008 | 0.1992 |
| **Residual** |  | 1.237E-7 | . |

| **Solutions for Fixed Effects** | | | | | | | |
| --- | --- | --- | --- | --- | --- | --- | --- |
| **Effect** | **Treatment** | **Year** | **Estimate** | **Standard Error** | **DF** | **t Value** | **Pr > \|t\|** |
| **Intercept** |  |  | 3.1432 | 1.0383 | 5.851 | 3.03 | 0.0239 |
| **Year** |  | 1999 | 1.2184 | 0.9684 | 11.28 | 1.26 | 0.2337 |
| **Year** |  | 2002 | 0.8718 | 0.8854 | 13.95 | 0.98 | 0.3416 |
| **Year** |  | 2009 | 1.3973 | 0.7620 | 15.87 | 1.83 | 0.0855 |
| **Year** |  | 2016 | 2.1441 | 0.5666 | 15.31 | 3.78 | 0.0017 |
| **Year** |  | 2019 | 0 | . | . | . | . |
| **Treatment** | Control |  | 1.0878 | 0.9152 | 2.044 | 1.19 | 0.3543 |
| **Treatment** | Exclosure |  | 0 | . | . | . | . |
| **Treatment*Year** | Control | 1999 | -1.0825 | 1.3695 | 11.28 | -0.79 | 0.4456 |
| **Treatment*Year** | Control | 2002 | -1.0139 | 1.2521 | 13.95 | -0.81 | 0.4317 |
| **Treatment*Year** | Control | 2009 | -1.0835 | 1.0776 | 15.87 | -1.01 | 0.3298 |
| **Treatment*Year** | Control | 2016 | -1.7613 | 0.8013 | 15.31 | -2.20 | 0.0437 |
| **Treatment*Year** | Control | 2019 | 0 | . | . | . | . |
| **Treatment*Year** | Exclosure | 1999 | 0 | . | . | . | . |
| **Treatment*Year** | Exclosure | 2002 | 0 | . | . | . | . |
| **Treatment*Year** | Exclosure | 2009 | 0 | . | . | . | . |
| **Treatment*Year** | Exclosure | 2016 | 0 | . | . | . | . |
| **Treatment*Year** | Exclosure | 2019 | 0 | . | . | . | . |

| **Type III Tests of Fixed Effects** | | | | |
| --- | --- | --- | --- | --- |
| **Effect** | **Num DF** | **Den DF** | **F Value** | **Pr > F** |
| **Year** | 4 | 14.09 | 3.01 | 0.0545 |
| **Treatment** | 1 | 1.19 | 0.03 | 0.8967 |
| **Treatment*Year** | 4 | 14.09 | 1.30 | 0.3173 |

| **Treatment*Year Least Squares Means** | | | | | | |
| --- | --- | --- | --- | --- | --- | --- |
| **Treatment** | **Year** | **Estimate** | **Standard Error** | **DF** | **t Value** | **Pr > \|t\|** |
| Control | 1999 | 4.3669 | 1.0383 | 5.851 | 4.21 | 0.0060 |
| Control | 2002 | 4.0889 | 1.0383 | 5.851 | 3.94 | 0.0080 |
| Control | 2009 | 4.5449 | 1.0383 | 5.851 | 4.38 | 0.0050 |
| Control | 2016 | 4.6139 | 1.0383 | 5.851 | 4.44 | 0.0046 |
| Control | 2019 | 4.2310 | 1.0383 | 5.851 | 4.08 | 0.0069 |
| Exclosure | 1999 | 4.3616 | 1.0383 | 5.851 | 4.20 | 0.0060 |
| Exclosure | 2002 | 4.0150 | 1.0383 | 5.851 | 3.87 | 0.0087 |
| Exclosure | 2009 | 4.5405 | 1.0383 | 5.851 | 4.37 | 0.0050 |
| Exclosure | 2016 | 5.2874 | 1.0383 | 5.851 | 5.09 | 0.0024 |
| Exclosure | 2019 | 3.1432 | 1.0383 | 5.851 | 3.03 | 0.0239 |

| **Tests of Effect Slices for Treatment*Year Sliced By Year** | | | | |
| --- | --- | --- | --- | --- |
| **Year** | **Num DF** | **Den DF** | **F Value** | **Pr > F** |
| 1999 | 1 | 2.044 | 0.00 | 0.9959 |
| 2002 | 1 | 2.044 | 0.01 | 0.9429 |
| 2009 | 1 | 2.044 | 0.00 | 0.9966 |
| 2016 | 1 | 2.044 | 0.54 | 0.5369 |
| 2019 | 1 | 2.044 | 1.41 | 0.3543 |

**(iii) Height growth**

| **Model Information** | |
| --- | --- |
| **Data Set** | SASUSER.MPALAEXCL_HEIGHT_GROWTH |
| **Response Variable** | Height_Growth |
| **Response Distribution** | Gaussian |
| **Link Function** | Identity |
| **Variance Function** | Default |
| **Variance Matrix** | Not blocked |
| **Estimation Technique** | Restricted Maximum Likelihood |
| **Degrees of Freedom Method** | Kenward-Roger |
| **Fixed Effects SE Adjustment** | Kenward-Roger |

| **Class Level Information** | | |
| --- | --- | --- |
| **Class** | **Levels** | **Values** |
| **Treatment** | 2 | Control Exclosure |
| **Block** | 3 | Baboon Kopi Mukenya |
| **Subject** | 6 | 1 2 3 4 5 6 |
| **Year** | 4 | 2002 2009 2016 2019 |

| **Number of Observations Read** | 24 |
| --- | --- |
| **Number of Observations Used** | 24 |

| **Dimensions** | |
| --- | --- |
| **G-side Cov. Parameters** | 3 |
| **R-side Cov. Parameters** | 1 |
| **Columns in X** | 15 |
| **Columns in Z** | 27 |
| **Subjects (Blocks in V)** | 1 |
| **Max Obs per Subject** | 24 |

| **Optimization Information** | |
| --- | --- |
| **Optimization Technique** | Dual Quasi-Newton |
| **Parameters in Optimization** | 3 |
| **Lower Boundaries** | 2 |
| **Upper Boundaries** | 0 |
| **Fixed Effects** | Profiled |
| **Residual Variance** | Profiled |
| **Starting From** | Data |

| **Iteration History** | | | | | |
| --- | --- | --- | --- | --- | --- |
| **Iteration** | **Restarts** | **Evaluations** | **Objective Function** | **Change** | **Max Gradient** |
| **0** | **0** | 4 | -55.92376411 | . | 5.095019 |
| **1** | **0** | 18 | -58.66983081 | 2.74606670 | 2.987621 |
| **2** | **0** | 5 | -58.67429632 | 0.00446551 | 4.131369 |
| **3** | **0** | 5 | -58.89617582 | 0.22187950 | 1.562869 |
| **4** | **0** | 2 | -58.89673837 | 0.00056255 | 0.599577 |
| **5** | **0** | 2 | -58.89682939 | 0.00009102 | 0.103867 |
| **6** | **0** | 3 | -58.89683623 | 0.00000684 | 0.002824 |
| **7** | **0** | 3 | -58.89683626 | 0.00000003 | 0.000856 |

| Convergence criterion (GCONV=1E-8) satisfied. |
| --- |

| **Estimated G matrix is not positive definite.** |
| --- |

| **Fit Statistics** | |
| --- | --- |
| **-2 Res Log Likelihood** | -58.90 |
| **AIC (smaller is better)** | -50.90 |
| **AICC (smaller is better)** | -47.26 |
| **BIC (smaller is better)** | -54.50 |
| **CAIC (smaller is better)** | -50.50 |
| **HQIC (smaller is better)** | -58.14 |
| **Generalized Chi-Square** | 0.01 |
| **Gener. Chi-Square / DF** | 0.00 |

| **Covariance Parameter Estimates** | | | |
| --- | --- | --- | --- |
| **Cov Parm** | **Subject** | **Estimate** | **Standard Error** |
| **Block** |  | 0.000157 | 0.000170 |
| **Variance** | Subject | 0.000254 | 0.000459 |
| **CS** | Subject | -0.00026 | 0.000118 |
| **Residual** |  | 0.000871 | . |

| **Solutions for Fixed Effects** | | | | | | | |
| --- | --- | --- | --- | --- | --- | --- | --- |
| **Effect** | **Treatment** | **Year** | **Estimate** | **Standard Error** | **DF** | **t Value** | **Pr > \|t\|** |
| **Intercept** |  |  | -0.2696 | 0.01849 | 14.27 | -14.58 | <.0001 |
| **Year** |  | 2002 | 0.3537 | 0.02739 | 12 | 12.91 | <.0001 |
| **Year** |  | 2009 | 0.4198 | 0.02739 | 12 | 15.33 | <.0001 |
| **Year** |  | 2016 | 0.2958 | 0.02739 | 12 | 10.80 | <.0001 |
| **Year** |  | 2019 | 0 | . | . | . | . |
| **Treatment** | Control |  | 0.2399 | 0.02407 | 12.66 | 9.97 | <.0001 |
| **Treatment** | Exclosure |  | 0 | . | . | . | . |
| **Treatment*Year** | Control | 2002 | -0.3792 | 0.03874 | 12 | -9.79 | <.0001 |
| **Treatment*Year** | Control | 2009 | -0.3911 | 0.03874 | 12 | -10.10 | <.0001 |
| **Treatment*Year** | Control | 2016 | -0.2578 | 0.03874 | 12 | -6.66 | <.0001 |
| **Treatment*Year** | Control | 2019 | 0 | . | . | . | . |
| **Treatment*Year** | Exclosure | 2002 | 0 | . | . | . | . |
| **Treatment*Year** | Exclosure | 2009 | 0 | . | . | . | . |
| **Treatment*Year** | Exclosure | 2016 | 0 | . | . | . | . |
| **Treatment*Year** | Exclosure | 2019 | 0 | . | . | . | . |

| **Type III Tests of Fixed Effects** | | | | |
| --- | --- | --- | --- | --- |
| **Effect** | **Num DF** | **Den DF** | **F Value** | **Pr > F** |
| **Year** | 3 | 12 | 49.74 | <.0001 |
| **Treatment** | 1 | 2 | 17.39 | 0.0530 |
| **Treatment*Year** | 3 | 12 | 43.97 | <.0001 |

| **Treatment*Year Least Squares Means** | | | | | | |
| --- | --- | --- | --- | --- | --- | --- |
| **Treatment** | **Year** | **Estimate** | **Standard Error** | **DF** | **t Value** | **Pr > \|t\|** |
| Control | 2002 | -0.05523 | 0.01849 | 14.27 | -2.99 | 0.0096 |
| Control | 2009 | -0.00100 | 0.01849 | 14.27 | -0.05 | 0.9576 |
| Control | 2016 | 0.008302 | 0.01849 | 14.27 | 0.45 | 0.6602 |
| Control | 2019 | -0.02968 | 0.01849 | 14.27 | -1.60 | 0.1304 |
| Exclosure | 2002 | 0.08403 | 0.01849 | 14.27 | 4.54 | 0.0004 |
| Exclosure | 2009 | 0.1502 | 0.01849 | 14.27 | 8.12 | <.0001 |
| Exclosure | 2016 | 0.02617 | 0.01849 | 14.27 | 1.42 | 0.1785 |
| Exclosure | 2019 | -0.2696 | 0.01849 | 14.27 | -14.58 | <.0001 |

| **Tests of Effect Slices for Treatment*Year Sliced By Year** | | | | |
| --- | --- | --- | --- | --- |
| **Year** | **Num DF** | **Den DF** | **F Value** | **Pr > F** |
| 2002 | 1 | 12.66 | 33.47 | <.0001 |
| 2009 | 1 | 12.66 | 39.43 | <.0001 |
| 2016 | 1 | 12.66 | 0.55 | 0.4715 |
| 2019 | 1 | 12.66 | 99.35 | <.0001 |

**(iv) Canopy area growth**

| **Model Information** | |
| --- | --- |
| **Data Set** | SASUSER.MPALAEXCL_AREA_GROWTH |
| **Response Variable** | Growth |
| **Response Distribution** | Gaussian |
| **Link Function** | Identity |
| **Variance Function** | Default |
| **Variance Matrix** | Not blocked |
| **Estimation Technique** | Restricted Maximum Likelihood |
| **Degrees of Freedom Method** | Kenward-Roger |
| **Fixed Effects SE Adjustment** | Kenward-Roger |

| **Class Level Information** | | |
| --- | --- | --- |
| **Class** | **Levels** | **Values** |
| **Treatment** | 2 | Control Exclosure |
| **Block** | 3 | Baboon Kopi Mukenya |
| **Subject** | 6 | 1 2 3 4 5 6 |
| **Year** | 4 | 2002 2009 2016 2019 |

| **Number of Observations Read** | 24 |
| --- | --- |
| **Number of Observations Used** | 24 |

| **Dimensions** | |
| --- | --- |
| **G-side Cov. Parameters** | 3 |
| **R-side Cov. Parameters** | 1 |
| **Columns in X** | 15 |
| **Columns in Z** | 27 |
| **Subjects (Blocks in V)** | 1 |
| **Max Obs per Subject** | 24 |

| **Optimization Information** | |
| --- | --- |
| **Optimization Technique** | Dual Quasi-Newton |
| **Parameters in Optimization** | 3 |
| **Lower Boundaries** | 3 |
| **Upper Boundaries** | 1 |
| **Fixed Effects** | Profiled |
| **Residual Variance** | Profiled |
| **Starting From** | Data |

| **Iteration History** | | | | | |
| --- | --- | --- | --- | --- | --- |
| **Iteration** | **Restarts** | **Evaluations** | **Objective Function** | **Change** | **Max Gradient** |
| **0** | **0** | 4 | -28.00938225 | . | 14.75557 |
| **1** | **0** | 3 | -28.6157162 | 0.60633395 | 1.255851 |
| **2** | **0** | 4 | -28.7756286 | 0.15991240 | 5.621974 |
| **3** | **0** | 4 | -29.79781878 | 1.02219018 | 12.21896 |
| **4** | **0** | 2 | -29.95698569 | 0.15916691 | 8.632895 |
| **5** | **0** | 4 | -31.00038294 | 1.04339724 | 3.000083 |
| **6** | **0** | 2 | -31.24586782 | 0.24548489 | 1.099815 |
| **7** | **0** | 3 | -31.25624497 | 0.01037715 | 0.561497 |
| **8** | **0** | 4 | -31.29664975 | 0.04040478 | 1.13094 |
| **9** | **0** | 2 | -31.36128761 | 0.06463785 | 0.856012 |
| **10** | **0** | 4 | -31.47815451 | 0.11686690 | 1.429386 |
| **11** | **0** | 3 | -31.55087127 | 0.07271676 | 0.52163 |
| **12** | **0** | 2 | -31.64718127 | 0.09631000 | 0.365889 |
| **13** | **0** | 3 | -31.66460136 | 0.01742009 | 0.29812 |
| **14** | **0** | 2 | -31.68402853 | 0.01942717 | 0.303042 |
| **15** | **0** | 3 | -31.68608149 | 0.00205296 | 0.02683 |
| **16** | **0** | 3 | -31.68711122 | 0.00102973 | 0.052837 |
| **17** | **0** | 2 | -31.68762156 | 0.00051034 | 0.161758 |
| **18** | **0** | 4 | -31.68929708 | 0.00167552 | 0.100528 |
| **19** | **0** | 2 | -31.69080048 | 0.00150340 | 0.026066 |
| **20** | **0** | 3 | -31.69111932 | 0.00031884 | 0.035515 |
| **21** | **0** | 3 | -31.69115552 | 0.00003619 | 0.004636 |
| **22** | **0** | 3 | -31.69116049 | 0.00000497 | 0.001395 |
| **23** | **0** | 3 | -31.69116054 | 0.00000005 | 0.000034 |

| Convergence criterion (GCONV=1E-8) satisfied. |
| --- |

| **Fit Statistics** | |
| --- | --- |
| **-2 Res Log Likelihood** | -31.69 |
| **AIC (smaller is better)** | -23.69 |
| **AICC (smaller is better)** | -20.05 |
| **BIC (smaller is better)** | -27.30 |
| **CAIC (smaller is better)** | -23.30 |
| **HQIC (smaller is better)** | -30.94 |
| **Generalized Chi-Square** | 0.01 |
| **Gener. Chi-Square / DF** | 0.00 |

| **Covariance Parameter Estimates** | | | |
| --- | --- | --- | --- |
| **Cov Parm** | **Subject** | **Estimate** | **Standard Error** |
| **Block** |  | 0.001679 | 0.001992 |
| **Variance** | Subject | 0.003856 | 0.003821 |
| **AR(1)** | Subject | -0.6260 | 0.5104 |
| **Residual** |  | 0.000728 | 0.003479 |

| **Solutions for Fixed Effects** | | | | | | | |
| --- | --- | --- | --- | --- | --- | --- | --- |
| **Effect** | **Treatment** | **Year** | **Estimate** | **Standard Error** | **DF** | **t Value** | **Pr > \|t\|** |
| **Intercept** |  |  | -0.7567 | 0.04569 | 9.732 | -16.56 | <.0001 |
| **Year** |  | 2002 | 1.1668 | 0.05818 | 5.139 | 20.06 | <.0001 |
| **Year** |  | 2009 | 1.1992 | 0.04125 | 8.35 | 29.08 | <.0001 |
| **Year** |  | 2016 | 0.8446 | 0.07402 | 6.913 | 11.41 | <.0001 |
| **Year** |  | 2019 | 0 | . | . | . | . |
| **Treatment** | Control |  | 0.5907 | 0.05528 | 10.4 | 10.68 | <.0001 |
| **Treatment** | Exclosure |  | 0 | . | . | . | . |
| **Treatment*Year** | Control | 2002 | -1.0156 | 0.08228 | 5.139 | -12.34 | <.0001 |
| **Treatment*Year** | Control | 2009 | -0.9756 | 0.05833 | 8.35 | -16.73 | <.0001 |
| **Treatment*Year** | Control | 2016 | -0.6157 | 0.1047 | 6.913 | -5.88 | 0.0006 |
| **Treatment*Year** | Control | 2019 | 0 | . | . | . | . |
| **Treatment*Year** | Exclosure | 2002 | 0 | . | . | . | . |
| **Treatment*Year** | Exclosure | 2009 | 0 | . | . | . | . |
| **Treatment*Year** | Exclosure | 2016 | 0 | . | . | . | . |
| **Treatment*Year** | Exclosure | 2019 | 0 | . | . | . | . |

| **Type III Tests of Fixed Effects** | | | | |
| --- | --- | --- | --- | --- |
| **Effect** | **Num DF** | **Den DF** | **F Value** | **Pr > F** |
| **Year** | 3 | 7.111 | 492.32 | <.0001 |
| **Treatment** | 1 | 4.701 | 24.30 | 0.0052 |
| **Treatment*Year** | 3 | 7.111 | 309.50 | <.0001 |

| **Treatment*Year Least Squares Means** | | | | | | |
| --- | --- | --- | --- | --- | --- | --- |
| **Treatment** | **Year** | **Estimate** | **Standard Error** | **DF** | **t Value** | **Pr > \|t\|** |
| Control | 2002 | -0.01486 | 0.04569 | 9.732 | -0.33 | 0.7519 |
| Control | 2009 | 0.05764 | 0.04569 | 9.732 | 1.26 | 0.2365 |
| Control | 2016 | 0.06286 | 0.04569 | 9.732 | 1.38 | 0.1998 |
| Control | 2019 | -0.1660 | 0.04569 | 9.732 | -3.63 | 0.0048 |
| Exclosure | 2002 | 0.4101 | 0.04569 | 9.732 | 8.98 | <.0001 |
| Exclosure | 2009 | 0.4426 | 0.04569 | 9.732 | 9.69 | <.0001 |
| Exclosure | 2016 | 0.08794 | 0.04569 | 9.732 | 1.92 | 0.0840 |
| Exclosure | 2019 | -0.7567 | 0.04569 | 9.732 | -16.56 | <.0001 |

| **Tests of Effect Slices for Treatment*Year Sliced By Year** | | | | |
| --- | --- | --- | --- | --- |
| **Year** | **Num DF** | **Den DF** | **F Value** | **Pr > F** |
| 2002 | 1 | 10.4 | 59.09 | <.0001 |
| 2009 | 1 | 10.4 | 48.48 | <.0001 |
| 2016 | 1 | 10.4 | 0.21 | 0.6594 |
| 2019 | 1 | 10.4 | 114.14 | <.0001 |

**(v) Tree density**

| **Model Information** | |
| --- | --- |
| **Data Set** | SASUSER.MPALAEXCL_DENSITY |
| **Response Variable** | Density |
| **Response Distribution** | Gaussian |
| **Link Function** | Identity |
| **Variance Function** | Default |
| **Variance Matrix** | Not blocked |
| **Estimation Technique** | Restricted Maximum Likelihood |
| **Degrees of Freedom Method** | Kenward-Roger |
| **Fixed Effects SE Adjustment** | Kenward-Roger |

| **Class Level Information** | | |
| --- | --- | --- |
| **Class** | **Levels** | **Values** |
| **Treatment** | 2 | Control Exclosure |
| **Block** | 3 | Baboon Kopi Mukenya |
| **Subject** | 6 | 1 2 3 4 5 6 |
| **Year** | 5 | 1999 2002 2009 2016 2019 |

| **Number of Observations Read** | 30 |
| --- | --- |
| **Number of Observations Used** | 30 |

| **Dimensions** | |
| --- | --- |
| **G-side Cov. Parameters** | 3 |
| **R-side Cov. Parameters** | 1 |
| **Columns in X** | 18 |
| **Columns in Z** | 33 |
| **Subjects (Blocks in V)** | 1 |
| **Max Obs per Subject** | 30 |

| **Optimization Information** | |
| --- | --- |
| **Optimization Technique** | Dual Quasi-Newton |
| **Parameters in Optimization** | 3 |
| **Lower Boundaries** | 3 |
| **Upper Boundaries** | 1 |
| **Fixed Effects** | Profiled |
| **Residual Variance** | Profiled |
| **Starting From** | Data |

| **Iteration History** | | | | | |
| --- | --- | --- | --- | --- | --- |
| **Iteration** | **Restarts** | **Evaluations** | **Objective Function** | **Change** | **Max Gradient** |
| **0** | **0** | 4 | 239.03309066 | . | 26.6328 |
| **1** | **0** | 4 | 226.274633 | 12.75845766 | 22.77805 |
| **2** | **0** | 9 | 221.49136846 | 4.78326454 | 8.205147 |
| **3** | **0** | 3 | 221.25698695 | 0.23438150 | 2.828543 |
| **4** | **0** | 2 | 221.23470314 | 0.02228382 | 0.913453 |
| **5** | **0** | 2 | 221.23250655 | 0.00219659 | 0.076683 |
| **6** | **0** | 3 | 221.23249037 | 0.00001618 | 0.000916 |
| **7** | **0** | 3 | 221.23249036 | 0.00000000 | 0.000381 |

| Convergence criterion (GCONV=1E-8) satisfied. |
| --- |

| **Fit Statistics** | |
| --- | --- |
| **-2 Res Log Likelihood** | 221.23 |
| **AIC (smaller is better)** | 229.23 |
| **AICC (smaller is better)** | 231.90 |
| **BIC (smaller is better)** | 225.63 |
| **CAIC (smaller is better)** | 229.63 |
| **HQIC (smaller is better)** | 221.98 |
| **Generalized Chi-Square** | 24.17 |
| **Gener. Chi-Square / DF** | 1.21 |

| **Covariance Parameter Estimates** | | | |
| --- | --- | --- | --- |
| **Cov Parm** | **Subject** | **Estimate** | **Standard Error** |
| **Block** | Â | 41048 | 52047 |
| **Variance** | Subject | 3980.57 | 2573.77 |
| **AR(1)** | Subject | 0.8344 | 0.1143 |
| **Residual** | Â | 1.2084 | . |

| **Solutions for Fixed Effects** | | | | | | | |
| --- | --- | --- | --- | --- | --- | --- | --- |
| **Effect** | **Treatment** | **Year** | **Estimate** | **Standard Error** | **DF** | **tÂ Value** | **Pr > \|t\|** |
| **Intercept** | Â | Â | 718.00 | 122.52 | 1.496 | 5.86 | 0.0523 |
| **Year** | Â | 1999 | -332.00 | 43.6552 | 18.36 | -7.61 | <.0001 |
| **Year** | Â | 2002 | -225.67 | 39.4049 | 18.65 | -5.73 | <.0001 |
| **Year** | Â | 2009 | 103.33 | 33.5221 | 17.86 | 3.08 | 0.0065 |
| **Year** | Â | 2016 | 54.6667 | 24.6847 | 16.08 | 2.21 | 0.0416 |
| **Year** | Â | 2019 | 0 | . | . | . | . |
| **Treatment** | Control | Â | -391.67 | 51.5221 | 4.787 | -7.60 | 0.0008 |
| **Treatment** | Exclosure | Â | 0 | . | . | . | . |
| **Treatment*Year** | Control | 1999 | 318.33 | 61.7377 | 18.36 | 5.16 | <.0001 |
| **Treatment*Year** | Control | 2002 | 220.00 | 55.7270 | 18.65 | 3.95 | 0.0009 |
| **Treatment*Year** | Control | 2009 | -105.33 | 47.4074 | 17.86 | -2.22 | 0.0395 |
| **Treatment*Year** | Control | 2016 | -32.0000 | 34.9094 | 16.08 | -0.92 | 0.3729 |
| **Treatment*Year** | Control | 2019 | 0 | . | . | . | . |
| **Treatment*Year** | Exclosure | 1999 | 0 | . | . | . | . |
| **Treatment*Year** | Exclosure | 2002 | 0 | . | . | . | . |
| **Treatment*Year** | Exclosure | 2009 | 0 | . | . | . | . |
| **Treatment*Year** | Exclosure | 2016 | 0 | . | . | . | . |
| **Treatment*Year** | Exclosure | 2019 | 0 | . | . | . | . |

| **Type III Tests of Fixed Effects** | | | | |
| --- | --- | --- | --- | --- |
| **Effect** | **Num DF** | **Den DF** | **F Value** | **PrÂ >Â F** |
| **Year** | 4 | 15.87 | 27.05 | <.0001 |
| **Treatment** | 1 | 3.037 | 54.23 | 0.0050 |
| **Treatment*Year** | 4 | 15.87 | 25.05 | <.0001 |

| **Treatment*Year Least Squares Means** | | | | | | |
| --- | --- | --- | --- | --- | --- | --- |
| **Treatment** | **Year** | **Estimate** | **Standard Error** | **DF** | **tÂ Value** | **Pr > \|t\|** |
| Control | 1999 | 312.67 | 122.52 | 1.496 | 2.55 | 0.1658 |
| Control | 2002 | 320.67 | 122.52 | 1.496 | 2.62 | 0.1605 |
| Control | 2009 | 324.33 | 122.52 | 1.496 | 2.65 | 0.1581 |
| Control | 2016 | 349.00 | 122.52 | 1.496 | 2.85 | 0.1436 |
| Control | 2019 | 326.33 | 122.52 | 1.496 | 2.66 | 0.1568 |
| Exclosure | 1999 | 386.00 | 122.52 | 1.496 | 3.15 | 0.1254 |
| Exclosure | 2002 | 492.33 | 122.52 | 1.496 | 4.02 | 0.0896 |
| Exclosure | 2009 | 821.33 | 122.52 | 1.496 | 6.70 | 0.0430 |
| Exclosure | 2016 | 772.67 | 122.52 | 1.496 | 6.31 | 0.0470 |
| Exclosure | 2019 | 718.00 | 122.52 | 1.496 | 5.86 | 0.0523 |

| **Tests of Effect Slices for Treatment*Year Sliced By Year** | | | | |
| --- | --- | --- | --- | --- |
| **Year** | **Num DF** | **Den DF** | **F Value** | **PrÂ >Â F** |
| 1999 | 1 | 4.787 | 2.03 | 0.2164 |
| 2002 | 1 | 4.787 | 11.10 | 0.0221 |
| 2009 | 1 | 4.787 | 93.05 | 0.0003 |
| 2016 | 1 | 4.787 | 67.62 | 0.0005 |
| 2019 | 1 | 4.787 | 57.79 | 0.0008 |

**(vi) Tree recruitment**

| **Model Information** | |
| --- | --- |
| **Data Set** | SASUSER.MPALAEXCL_RECRUITMENT |
| **Response Variable** | Recruitment |
| **Response Distribution** | Gaussian |
| **Link Function** | Identity |
| **Variance Function** | Default |
| **Variance Matrix** | Not blocked |
| **Estimation Technique** | Restricted Maximum Likelihood |
| **Degrees of Freedom Method** | Kenward-Roger |
| **Fixed Effects SE Adjustment** | Kenward-Roger |

| **Class Level Information** | | |
| --- | --- | --- |
| **Class** | **Levels** | **Values** |
| **Treatment** | 2 | Control Exclosure |
| **Block** | 3 | Baboon Kopi Mukenya |
| **Subject** | 6 | 1 2 3 4 5 6 |
| **Year** | 4 | 2002 2009 2016 2019 |

| **Number of Observations Read** | 24 |
| --- | --- |
| **Number of Observations Used** | 24 |

| **Dimensions** | |
| --- | --- |
| **G-side Cov. Parameters** | 3 |
| **R-side Cov. Parameters** | 1 |
| **Columns in X** | 15 |
| **Columns in Z** | 27 |
| **Subjects (Blocks in V)** | 1 |
| **Max Obs per Subject** | 24 |

| **Optimization Information** | |
| --- | --- |
| **Optimization Technique** | Dual Quasi-Newton |
| **Parameters in Optimization** | 3 |
| **Lower Boundaries** | 2 |
| **Upper Boundaries** | 0 |
| **Fixed Effects** | Profiled |
| **Residual Variance** | Profiled |
| **Starting From** | Data |

| **Iteration History** | | | | | |
| --- | --- | --- | --- | --- | --- |
| **Iteration** | **Restarts** | **Evaluations** | **Objective Function** | **Change** | **Max Gradient** |
| **0** | **0** | 4 | 116.36143732 | . | 0.001875 |
| **1** | **0** | 4 | 116.36094083 | 0.00049649 | 0.00069 |
| **2** | **0** | 2 | 116.36088414 | 0.00005669 | 0.000186 |
| **3** | **0** | 4 | 116.3608726 | 0.00001154 | 0.000183 |
| **4** | **0** | 2 | 116.36086944 | 0.00000316 | 0.000059 |
| **5** | **0** | 2 | 116.36086907 | 0.00000037 | 1.982E-7 |

| Convergence criterion (GCONV=1E-8) satisfied. |
| --- |

| **Fit Statistics** | |
| --- | --- |
| **-2 Res Log Likelihood** | 116.36 |
| **AIC (smaller is better)** | 124.36 |
| **AICC (smaller is better)** | 128.00 |
| **BIC (smaller is better)** | 120.76 |
| **CAIC (smaller is better)** | 124.76 |
| **HQIC (smaller is better)** | 117.11 |
| **Generalized Chi-Square** | 15.64 |
| **Gener. Chi-Square / DF** | 0.98 |

| **Covariance Parameter Estimates** | | | |
| --- | --- | --- | --- |
| **Cov Parm** | **Subject** | **Estimate** | **Standard Error** |
| **Block** |  | 6.0876 | 22.5858 |
| **Variance** | Subject | 34.7645 | 14.5917 |
| **CS** | Subject | 16.3325 | 25.5300 |
| **Residual** |  | 0.9778 | . |

| **Solutions for Fixed Effects** | | | | | | | |
| --- | --- | --- | --- | --- | --- | --- | --- |
| **Effect** | **Treatment** | **Year** | **Estimate** | **Standard Error** | **DF** | **t Value** | **Pr > \|t\|** |
| **Intercept** |  |  | 12.3333 | 4.4031 | 10.74 | 2.80 | 0.0176 |
| **Year** |  | 2002 | 24.1111 | 4.8814 | 12 | 4.94 | 0.0003 |
| **Year** |  | 2009 | 37.8571 | 4.8814 | 12 | 7.76 | <.0001 |
| **Year** |  | 2016 | -0.9524 | 4.8814 | 12 | -0.20 | 0.8486 |
| **Year** |  | 2019 | 0 | . | . | . | . |
| **Treatment** | Control |  | -5.8889 | 5.8921 | 7.153 | -1.00 | 0.3502 |
| **Treatment** | Exclosure |  | 0 | . | . | . | . |
| **Treatment*Year** | Control | 2002 | -23.6667 | 6.9034 | 12 | -3.43 | 0.0050 |
| **Treatment*Year** | Control | 2009 | -37.0635 | 6.9034 | 12 | -5.37 | 0.0002 |
| **Treatment*Year** | Control | 2016 | 7.8889 | 6.9034 | 12 | 1.14 | 0.2754 |
| **Treatment*Year** | Control | 2019 | 0 | . | . | . | . |
| **Treatment*Year** | Exclosure | 2002 | 0 | . | . | . | . |
| **Treatment*Year** | Exclosure | 2009 | 0 | . | . | . | . |
| **Treatment*Year** | Exclosure | 2016 | 0 | . | . | . | . |
| **Treatment*Year** | Exclosure | 2019 | 0 | . | . | . | . |

| **Type III Tests of Fixed Effects** | | | | |
| --- | --- | --- | --- | --- |
| **Effect** | **Num DF** | **Den DF** | **F Value** | **Pr > F** |
| **Year** | 3 | 12 | 13.09 | 0.0004 |
| **Treatment** | 1 | 2 | 21.65 | 0.0432 |
| **Treatment*Year** | 3 | 12 | 18.16 | <.0001 |

| **Treatment*Year Least Squares Means** | | | | | | |
| --- | --- | --- | --- | --- | --- | --- |
| **Treatment** | **Year** | **Estimate** | **Standard Error** | **DF** | **t Value** | **Pr > \|t\|** |
| Control | 2002 | 6.8889 | 4.4031 | 10.74 | 1.56 | 0.1467 |
| Control | 2009 | 7.2381 | 4.4031 | 10.74 | 1.64 | 0.1291 |
| Control | 2016 | 13.3810 | 4.4031 | 10.74 | 3.04 | 0.0116 |
| Control | 2019 | 6.4444 | 4.4031 | 10.74 | 1.46 | 0.1719 |
| Exclosure | 2002 | 36.4444 | 4.4031 | 10.74 | 8.28 | <.0001 |
| Exclosure | 2009 | 50.1905 | 4.4031 | 10.74 | 11.40 | <.0001 |
| Exclosure | 2016 | 11.3810 | 4.4031 | 10.74 | 2.58 | 0.0258 |
| Exclosure | 2019 | 12.3333 | 4.4031 | 10.74 | 2.80 | 0.0176 |

| **Tests of Effect Slices for Treatment*Year Sliced By Year** | | | | |
| --- | --- | --- | --- | --- |
| **Year** | **Num DF** | **Den DF** | **F Value** | **Pr > F** |
| 2002 | 1 | 7.153 | 25.16 | 0.0014 |
| 2009 | 1 | 7.153 | 53.14 | 0.0001 |
| 2016 | 1 | 7.153 | 0.12 | 0.7440 |
| 2019 | 1 | 7.153 | 1.00 | 0.3502 |

**(vii) Tree mortality**

| **Model Information** | |
| --- | --- |
| **Data Set** | SASUSER.MPALAEXCL_MORTALITY |
| **Response Variable** | Mortality |
| **Response Distribution** | Gaussian |
| **Link Function** | Identity |
| **Variance Function** | Default |
| **Variance Matrix** | Not blocked |
| **Estimation Technique** | Restricted Maximum Likelihood |
| **Degrees of Freedom Method** | Kenward-Roger |
| **Fixed Effects SE Adjustment** | Kenward-Roger |

| **Class Level Information** | | |
| --- | --- | --- |
| **Class** | **Levels** | **Values** |
| **Treatment** | 2 | Control Exclosure |
| **Block** | 3 | Baboon Kopi Mukenya |
| **Subject** | 6 | 1 2 3 4 5 6 |
| **Year** | 4 | 2002 2009 2016 2019 |

| **Number of Observations Read** | 24 |
| --- | --- |
| **Number of Observations Used** | 24 |

| **Dimensions** | |
| --- | --- |
| **G-side Cov. Parameters** | 3 |
| **R-side Cov. Parameters** | 1 |
| **Columns in X** | 15 |
| **Columns in Z** | 27 |
| **Subjects (Blocks in V)** | 1 |
| **Max Obs per Subject** | 24 |

| **Optimization Information** | |
| --- | --- |
| **Optimization Technique** | Dual Quasi-Newton |
| **Parameters in Optimization** | 3 |
| **Lower Boundaries** | 3 |
| **Upper Boundaries** | 1 |
| **Fixed Effects** | Profiled |
| **Residual Variance** | Profiled |
| **Starting From** | Data |

| **Iteration History** | | | | | |
| --- | --- | --- | --- | --- | --- |
| **Iteration** | **Restarts** | **Evaluations** | **Objective Function** | **Change** | **Max Gradient** |
| **0** | **0** | 4 | 102.22592635 | . | 4.531785 |
| **1** | **0** | 2 | 101.89195244 | 0.33397391 | 4.038937 |
| **2** | **0** | 2 | 101.50762699 | 0.38432545 | 0.127669 |
| **3** | **0** | 4 | 101.48788892 | 0.01973806 | 0.68707 |
| **4** | **0** | 4 | 101.45939301 | 0.02849592 | 1.203829 |
| **5** | **0** | 2 | 101.41304459 | 0.04634842 | 0.498346 |
| **6** | **0** | 3 | 101.4042583 | 0.00878629 | 0.201651 |
| **7** | **0** | 3 | 101.40322973 | 0.00102857 | 0.005875 |
| **8** | **0** | 3 | 101.40322628 | 0.00000345 | 0.002812 |
| **9** | **0** | 4 | 101.40319995 | 0.00002633 | 0.016861 |
| **10** | **0** | 2 | 101.40315467 | 0.00004528 | 0.000999 |
| **11** | **0** | 2 | 101.40308263 | 0.00007204 | 0.025339 |
| **12** | **0** | 4 | 101.40253283 | 0.00054980 | 0.118196 |
| **13** | **0** | 4 | 101.39577711 | 0.00675571 | 0.03853 |
| **14** | **0** | 3 | 101.39339275 | 0.00238437 | 0.216621 |
| **15** | **0** | 2 | 101.39049686 | 0.00289589 | 0.107379 |
| **16** | **0** | 2 | 101.38936997 | 0.00112689 | 0.303365 |
| **17** | **0** | 2 | 101.38754816 | 0.00182181 | 0.090895 |
| **18** | **0** | 3 | 101.38693676 | 0.00061140 | 0.064989 |
| **19** | **0** | 2 | 101.38602142 | 0.00091534 | 0.035826 |
| **20** | **0** | 2 | 101.38508079 | 0.00094064 | 0.04439 |
| **21** | **0** | 3 | 101.38490547 | 0.00017532 | 0.043096 |
| **22** | **0** | 2 | 101.38470443 | 0.00020104 | 0.038864 |
| **23** | **0** | 2 | 101.3846285 | 0.00007593 | 0.079103 |
| **24** | **0** | 2 | 101.3845082 | 0.00012030 | 0.021749 |
| **25** | **0** | 3 | 101.38446449 | 0.00004372 | 0.021537 |
| **26** | **0** | 2 | 101.38440139 | 0.00006310 | 0.01093 |
| **27** | **0** | 2 | 101.38433342 | 0.00006796 | 0.011566 |
| **28** | **0** | 3 | 101.38431801 | 0.00001542 | 0.014262 |
| **29** | **0** | 2 | 101.38430661 | 0.00001140 | 0.017269 |
| **30** | **0** | 2 | 101.38429946 | 0.00000715 | 0.013854 |
| **31** | **0** | 2 | 101.38429386 | 0.00000560 | 0.009691 |
| **32** | **0** | 2 | 101.3842907 | 0.00000316 | 0.011887 |
| **33** | **0** | 2 | 101.38428638 | 0.00000432 | 0.000945 |
| **34** | **0** | 2 | 101.38428037 | 0.00000601 | 0.004006 |
| **35** | **0** | 3 | 101.38427751 | 0.00000286 | 0.00598 |
| **36** | **0** | 3 | 101.38427567 | 0.00000184 | 0.002737 |
| **37** | **0** | 2 | 101.38427398 | 0.00000169 | 0.004743 |

| Convergence criterion (GCONV=1E-8) satisfied. |
| --- |

| **Fit Statistics** | |
| --- | --- |
| **-2 Res Log Likelihood** | 101.38 |
| **AIC (smaller is better)** | 109.38 |
| **AICC (smaller is better)** | 113.02 |
| **BIC (smaller is better)** | 105.78 |
| **CAIC (smaller is better)** | 109.78 |
| **HQIC (smaller is better)** | 102.14 |
| **Generalized Chi-Square** | 0.00 |
| **Gener. Chi-Square / DF** | 0.00 |

| **Covariance Parameter Estimates** | | | |
| --- | --- | --- | --- |
| **Cov Parm** | **Subject** | **Estimate** | **Standard Error** |
| **Block** |  | 1.7253 | 7.7714 |
| **Variance** | Subject | 20.2529 | 10.7583 |
| **AR(1)** | Subject | 0.3568 | 0.3853 |
| **Residual** |  | 0.000135 | . |

| **Solutions for Fixed Effects** | | | | | | | |
| --- | --- | --- | --- | --- | --- | --- | --- |
| **Effect** | **Treatment** | **Year** | **Estimate** | **Standard Error** | **DF** | **t Value** | **Pr > \|t\|** |
| **Intercept** |  |  | 30.5556 | 2.7067 | 12.27 | 11.29 | <.0001 |
| **Year** |  | 2002 | -29.5556 | 3.9718 | 10.47 | -7.44 | <.0001 |
| **Year** |  | 2009 | -27.3651 | 3.8950 | 13.65 | -7.03 | <.0001 |
| **Year** |  | 2016 | -12.2222 | 3.2594 | 10.29 | -3.75 | 0.0036 |
| **Year** |  | 2019 | 0 | . | . | . | . |
| **Treatment** | Control |  | -16.5556 | 3.6745 | 7.088 | -4.51 | 0.0027 |
| **Treatment** | Exclosure |  | 0 | . | . | . | . |
| **Treatment*Year** | Control | 2002 | 19.7778 | 5.6169 | 10.47 | 3.52 | 0.0052 |
| **Treatment*Year** | Control | 2009 | 20.0794 | 5.5084 | 13.65 | 3.65 | 0.0028 |
| **Treatment*Year** | Control | 2016 | 8.0794 | 4.6095 | 10.29 | 1.75 | 0.1093 |
| **Treatment*Year** | Control | 2019 | 0 | . | . | . | . |
| **Treatment*Year** | Exclosure | 2002 | 0 | . | . | . | . |
| **Treatment*Year** | Exclosure | 2009 | 0 | . | . | . | . |
| **Treatment*Year** | Exclosure | 2016 | 0 | . | . | . | . |
| **Treatment*Year** | Exclosure | 2019 | 0 | . | . | . | . |

| **Type III Tests of Fixed Effects** | | | | |
| --- | --- | --- | --- | --- |
| **Effect** | **Num DF** | **Den DF** | **F Value** | **Pr > F** |
| **Year** | 3 | 9.726 | 17.22 | 0.0003 |
| **Treatment** | 1 | 2.011 | 5.57 | 0.1415 |
| **Treatment*Year** | 3 | 9.726 | 5.05 | 0.0229 |

| **Treatment*Year Least Squares Means** | | | | | | |
| --- | --- | --- | --- | --- | --- | --- |
| **Treatment** | **Year** | **Estimate** | **Standard Error** | **DF** | **t Value** | **Pr > \|t\|** |
| Control | 2002 | 4.2222 | 2.7067 | 12.27 | 1.56 | 0.1442 |
| Control | 2009 | 6.7143 | 2.7067 | 12.27 | 2.48 | 0.0285 |
| Control | 2016 | 9.8571 | 2.7067 | 12.27 | 3.64 | 0.0033 |
| Control | 2019 | 14.0000 | 2.7067 | 12.27 | 5.17 | 0.0002 |
| Exclosure | 2002 | 1.0000 | 2.7067 | 12.27 | 0.37 | 0.7181 |
| Exclosure | 2009 | 3.1905 | 2.7067 | 12.27 | 1.18 | 0.2608 |
| Exclosure | 2016 | 18.3333 | 2.7067 | 12.27 | 6.77 | <.0001 |
| Exclosure | 2019 | 30.5556 | 2.7067 | 12.27 | 11.29 | <.0001 |

| **Tests of Effect Slices for Treatment*Year Sliced By Year** | | | | |
| --- | --- | --- | --- | --- |
| **Year** | **Num DF** | **Den DF** | **F Value** | **Pr > F** |
| 2002 | 1 | 7.088 | 0.77 | 0.4093 |
| 2009 | 1 | 7.088 | 0.92 | 0.3691 |
| 2016 | 1 | 7.088 | 5.32 | 0.0540 |
| 2019 | 1 | 7.088 | 20.30 | 0.0027 |

**(viii) Proportional canopy area**

| **Model Information** | |
| --- | --- |
| **Data Set** | SASUSER.MPALAEXCL_COMPOSITION |
| **Response Variable** | Percentage |
| **Response Distribution** | Gaussian |
| **Link Function** | Identity |
| **Variance Function** | Default |
| **Variance Matrix** | Not blocked |
| **Estimation Technique** | Restricted Maximum Likelihood |
| **Degrees of Freedom Method** | Kenward-Roger |
| **Fixed Effects SE Adjustment** | Kenward-Roger |

| **Class Level Information** | | |
| --- | --- | --- |
| **Class** | **Levels** | **Values** |
| **Treatment** | 2 | Control Exclosure |
| **Block** | 3 | Baboon Kopi Mukenya |
| **Subject** | 6 | 1 2 3 4 5 6 |
| **Spp** | 4 | Other etbc gten mel |
| **Year** | 5 | 1999 2002 2009 2016 2019 |

| **Number of Observations Read** | 120 |
| --- | --- |
| **Number of Observations Used** | 120 |

| **Dimensions** | |
| --- | --- |
| **G-side Cov. Parameters** | 16 |
| **R-side Cov. Parameters** | 1 |
| **Columns in X** | 90 |
| **Columns in Z** | 123 |
| **Subjects (Blocks in V)** | 1 |
| **Max Obs per Subject** | 120 |

| **Optimization Information** | |
| --- | --- |
| **Optimization Technique** | Dual Quasi-Newton |
| **Parameters in Optimization** | 16 |
| **Lower Boundaries** | 6 |
| **Upper Boundaries** | 0 |
| **Fixed Effects** | Profiled |
| **Residual Variance** | Profiled |
| **Starting From** | Data |

| **Iteration History** | | | | | |
| --- | --- | --- | --- | --- | --- |
| **Iteration** | **Restarts** | **Evaluations** | **Objective Function** | **Change** | **Max Gradient** |
| **0** | **0** | 4 | 469.53074216 | . | 7.62945 |
| **1** | **0** | 5 | 461.67439776 | 7.85634439 | 1.875187 |
| **2** | **0** | 6 | 461.48443494 | 0.18996283 | 3.022459 |
| **3** | **0** | 4 | 460.96636234 | 0.51807260 | 4.556942 |
| **4** | **0** | 2 | 460.72658083 | 0.23978151 | 2.538497 |
| **5** | **0** | 2 | 460.44044408 | 0.28613675 | 1.03313 |
| **6** | **0** | 3 | 460.37168606 | 0.06875803 | 0.471766 |
| **7** | **0** | 3 | 460.32788066 | 0.04380539 | 1.687202 |
| **8** | **0** | 2 | 460.26838188 | 0.05949878 | 0.299474 |
| **9** | **0** | 2 | 460.20651606 | 0.06186582 | 1.224676 |
| **10** | **0** | 3 | 460.18377834 | 0.02273772 | 0.184908 |
| **11** | **0** | 3 | 460.18112682 | 0.00265152 | 0.090685 |
| **12** | **0** | 2 | 460.17917644 | 0.00195038 | 0.153943 |
| **13** | **0** | 4 | 460.1725085 | 0.00666794 | 0.081547 |
| **14** | **0** | 2 | 460.16466159 | 0.00784691 | 0.128834 |
| **15** | **0** | 2 | 460.15437886 | 0.01028273 | 0.553092 |
| **16** | **0** | 4 | 460.12373678 | 0.03064208 | 0.173792 |
| **17** | **0** | 3 | 460.11857924 | 0.00515754 | 0.203439 |
| **18** | **0** | 4 | 460.06326839 | 0.05531084 | 1.413199 |
| **19** | **0** | 2 | 460.01259914 | 0.05066925 | 0.372293 |
| **20** | **0** | 3 | 459.98682781 | 0.02577134 | 0.212248 |
| **21** | **0** | 3 | 459.97529996 | 0.01152785 | 0.190423 |
| **22** | **0** | 3 | 459.97369209 | 0.00160787 | 0.116816 |
| **23** | **0** | 4 | 459.949235 | 0.02445709 | 0.692616 |
| **24** | **0** | 3 | 459.93242738 | 0.01680762 | 0.2886 |
| **25** | **0** | 2 | 459.9190621 | 0.01336527 | 0.091693 |
| **26** | **0** | 3 | 459.91619094 | 0.00287116 | 0.010896 |
| **27** | **0** | 3 | 459.9160733 | 0.00011764 | 0.01789 |
| **28** | **0** | 6 | 459.90483773 | 0.01123557 | 0.135779 |
| **29** | **0** | 3 | 459.89826646 | 0.00657126 | 0.025962 |
| **30** | **0** | 2 | 459.89603281 | 0.00223365 | 0.281482 |
| **31** | **0** | 4 | 459.89152734 | 0.00450547 | 0.169559 |
| **32** | **0** | 3 | 459.89021324 | 0.00131410 | 0.027262 |
| **33** | **0** | 3 | 459.88989264 | 0.00032060 | 0.058923 |
| **34** | **0** | 6 | 459.85665305 | 0.03323959 | 0.813116 |
| **35** | **0** | 3 | 459.85038006 | 0.00627299 | 0.061311 |
| **36** | **0** | 3 | 459.85005291 | 0.00032716 | 0.116615 |
| **37** | **0** | 4 | 459.84878209 | 0.00127081 | 0.031791 |
| **38** | **0** | 3 | 459.84873704 | 0.00004505 | 0.00163 |
| **39** | **0** | 3 | 459.84873576 | 0.00000128 | 0.001825 |

| Convergence criterion (GCONV=1E-8) satisfied. |
| --- |

| **Estimated G matrix is not positive definite.** |
| --- |

| **Fit Statistics** | |
| --- | --- |
| **-2 Res Log Likelihood** | 459.85 |
| **AIC (smaller is better)** | 491.85 |
| **AICC (smaller is better)** | 500.48 |
| **BIC (smaller is better)** | 477.43 |
| **CAIC (smaller is better)** | 493.43 |
| **HQIC (smaller is better)** | 462.86 |
| **Generalized Chi-Square** | 69.85 |
| **Gener. Chi-Square / DF** | 0.87 |

| **Covariance Parameter Estimates** | | | |
| --- | --- | --- | --- |
| **Cov Parm** | **Subject** | **Estimate** | **Standard Error** |
| **Block** |  | 0 | . |
| **UN(1,1)** | Spp(Subject) | 310.93 | 112.36 |
| **UN(2,1)** | Spp(Subject) | 277.11 | 100.55 |
| **UN(2,2)** | Spp(Subject) | 257.97 | 92.1841 |
| **UN(3,1)** | Spp(Subject) | 281.65 | 106.51 |
| **UN(3,2)** | Spp(Subject) | 277.62 | 100.00 |
| **UN(3,3)** | Spp(Subject) | 320.91 | 112.72 |
| **UN(4,1)** | Spp(Subject) | 288.98 | 110.25 |
| **UN(4,2)** | Spp(Subject) | 286.29 | 103.69 |
| **UN(4,3)** | Spp(Subject) | 333.47 | 117.13 |
| **UN(4,4)** | Spp(Subject) | 350.47 | 122.73 |
| **UN(5,1)** | Spp(Subject) | 298.40 | 113.45 |
| **UN(5,2)** | Spp(Subject) | 293.94 | 106.44 |
| **UN(5,3)** | Spp(Subject) | 341.08 | 120.01 |
| **UN(5,4)** | Spp(Subject) | 359.83 | 125.83 |
| **UN(5,5)** | Spp(Subject) | 368.69 | 129.19 |
| **Residual** |  | 0.8731 | . |

| **Solutions for Fixed Effects** | | | | | | | | |
| --- | --- | --- | --- | --- | --- | --- | --- | --- |
| **Effect** | **Treatment** | **Spp** | **Year** | **Estimate** | **Standard Error** | **DF** | **t Value** | **Pr > \|t\|** |
| **Intercept** |  |  |  | 25.1538 | 11.0989 | 16.37 | 2.27 | 0.0373 |
| **Year** |  |  | 1999 | -0.1960 | 5.3093 | 16.39 | -0.04 | 0.9710 |
| **Year** |  |  | 2002 | -1.0586 | 3.6751 | 16.76 | -0.29 | 0.7768 |
| **Year** |  |  | 2009 | 3.4616 | 1.7497 | 16.22 | 1.98 | 0.0651 |
| **Year** |  |  | 2016 | 1.8147 | 0.6416 | 15.88 | 2.83 | 0.0122 |
| **Year** |  |  | 2019 | 0 | . | . | . | . |
| **Treatment** | Control |  |  | -4.0114 | 15.6963 | 16.37 | -0.26 | 0.8015 |
| **Treatment** | Exclosure |  |  | 0 | . | . | . | . |
| **Treatment*Year** | Control |  | 1999 | 8.8033 | 7.5085 | 16.39 | 1.17 | 0.2578 |
| **Treatment*Year** | Control |  | 2002 | 9.4197 | 5.1973 | 16.76 | 1.81 | 0.0879 |
| **Treatment*Year** | Control |  | 2009 | 3.3497 | 2.4745 | 16.22 | 1.35 | 0.1944 |
| **Treatment*Year** | Control |  | 2016 | 0.2282 | 0.9074 | 15.88 | 0.25 | 0.8046 |
| **Treatment*Year** | Control |  | 2019 | 0 | . | . | . | . |
| **Treatment*Year** | Exclosure |  | 1999 | 0 | . | . | . | . |
| **Treatment*Year** | Exclosure |  | 2002 | 0 | . | . | . | . |
| **Treatment*Year** | Exclosure |  | 2009 | 0 | . | . | . | . |
| **Treatment*Year** | Exclosure |  | 2016 | 0 | . | . | . | . |
| **Treatment*Year** | Exclosure |  | 2019 | 0 | . | . | . | . |
| **Spp** |  | Other |  | 8.1472 | 15.6963 | 16.37 | 0.52 | 0.6107 |
| **Spp** |  | etbc |  | 0.6978 | 15.6963 | 16.37 | 0.04 | 0.9651 |
| **Spp** |  | gten |  | -9.4602 | 15.6963 | 16.37 | -0.60 | 0.5550 |
| **Spp** |  | mel |  | 0 | . | . | . | . |
| **Spp*Year** |  | Other | 1999 | 4.2896 | 7.5086 | 16.39 | 0.57 | 0.5755 |
| **Spp*Year** |  | Other | 2002 | 5.6850 | 5.1974 | 16.76 | 1.09 | 0.2895 |
| **Spp*Year** |  | Other | 2009 | -7.5936 | 2.4745 | 16.22 | -3.07 | 0.0073 |
| **Spp*Year** |  | Other | 2016 | -4.1664 | 0.9074 | 15.88 | -4.59 | 0.0003 |
| **Spp*Year** |  | Other | 2019 | 0 | . | . | . | . |
| **Spp*Year** |  | etbc | 1999 | -2.7245 | 7.5085 | 16.39 | -0.36 | 0.7213 |
| **Spp*Year** |  | etbc | 2002 | -3.2791 | 5.1974 | 16.76 | -0.63 | 0.5366 |
| **Spp*Year** |  | etbc | 2009 | -3.6506 | 2.4745 | 16.22 | -1.48 | 0.1593 |
| **Spp*Year** |  | etbc | 2016 | -2.1833 | 0.9074 | 15.88 | -2.41 | 0.0287 |
| **Spp*Year** |  | etbc | 2019 | 0 | . | . | . | . |
| **Spp*Year** |  | gten | 1999 | -0.7812 | 7.5086 | 16.39 | -0.10 | 0.9184 |
| **Spp*Year** |  | gten | 2002 | 1.8284 | 5.1974 | 16.76 | 0.35 | 0.7294 |
| **Spp*Year** |  | gten | 2009 | -2.6023 | 2.4745 | 16.22 | -1.05 | 0.3084 |
| **Spp*Year** |  | gten | 2016 | -0.9089 | 0.9074 | 15.88 | -1.00 | 0.3315 |
| **Spp*Year** |  | gten | 2019 | 0 | . | . | . | . |
| **Spp*Year** |  | mel | 1999 | 0 | . | . | . | . |
| **Spp*Year** |  | mel | 2002 | 0 | . | . | . | . |
| **Spp*Year** |  | mel | 2009 | 0 | . | . | . | . |
| **Spp*Year** |  | mel | 2016 | 0 | . | . | . | . |
| **Spp*Year** |  | mel | 2019 | 0 | . | . | . | . |
| **Treatment*Spp** | Control | Other |  | 6.9700 | 22.1979 | 16.37 | 0.31 | 0.7575 |
| **Treatment*Spp** | Control | etbc |  | 10.3962 | 22.1979 | 16.37 | 0.47 | 0.6457 |
| **Treatment*Spp** | Control | gten |  | -1.3205 | 22.1979 | 16.37 | -0.06 | 0.9533 |
| **Treatment*Spp** | Control | mel |  | 0 | . | . | . | . |
| **Treatment*Spp** | Exclosure | Other |  | 0 | . | . | . | . |
| **Treatment*Spp** | Exclosure | etbc |  | 0 | . | . | . | . |
| **Treatment*Spp** | Exclosure | gten |  | 0 | . | . | . | . |
| **Treatment*Spp** | Exclosure | mel |  | 0 | . | . | . | . |
| **Treatment*Spp*Year** | Control | Other | 1999 | -17.0530 | 10.6187 | 16.39 | -1.61 | 0.1274 |
| **Treatment*Spp*Year** | Control | Other | 2002 | -18.5500 | 7.3502 | 16.76 | -2.52 | 0.0220 |
| **Treatment*Spp*Year** | Control | Other | 2009 | -1.8263 | 3.4995 | 16.22 | -0.52 | 0.6088 |
| **Treatment*Spp*Year** | Control | Other | 2016 | 1.1825 | 1.2832 | 15.88 | 0.92 | 0.3706 |
| **Treatment*Spp*Year** | Control | Other | 2019 | 0 | . | . | . | . |
| **Treatment*Spp*Year** | Control | etbc | 1999 | -12.2325 | 10.6187 | 16.39 | -1.15 | 0.2659 |
| **Treatment*Spp*Year** | Control | etbc | 2002 | -10.7503 | 7.3502 | 16.76 | -1.46 | 0.1621 |
| **Treatment*Spp*Year** | Control | etbc | 2009 | -6.6667 | 3.4995 | 16.22 | -1.91 | 0.0747 |
| **Treatment*Spp*Year** | Control | etbc | 2016 | -1.3466 | 1.2832 | 15.88 | -1.05 | 0.3097 |
| **Treatment*Spp*Year** | Control | etbc | 2019 | 0 | . | . | . | . |
| **Treatment*Spp*Year** | Control | gten | 1999 | -5.9277 | 10.6187 | 16.39 | -0.56 | 0.5842 |
| **Treatment*Spp*Year** | Control | gten | 2002 | -8.3785 | 7.3502 | 16.76 | -1.14 | 0.2704 |
| **Treatment*Spp*Year** | Control | gten | 2009 | -4.9058 | 3.4995 | 16.22 | -1.40 | 0.1798 |
| **Treatment*Spp*Year** | Control | gten | 2016 | -0.7489 | 1.2832 | 15.88 | -0.58 | 0.5677 |
| **Treatment*Spp*Year** | Control | gten | 2019 | 0 | . | . | . | . |
| **Treatment*Spp*Year** | Control | mel | 1999 | 0 | . | . | . | . |
| **Treatment*Spp*Year** | Control | mel | 2002 | 0 | . | . | . | . |
| **Treatment*Spp*Year** | Control | mel | 2009 | 0 | . | . | . | . |
| **Treatment*Spp*Year** | Control | mel | 2016 | 0 | . | . | . | . |
| **Treatment*Spp*Year** | Control | mel | 2019 | 0 | . | . | . | . |
| **Treatment*Spp*Year** | Exclosure | Other | 1999 | 0 | . | . | . | . |
| **Treatment*Spp*Year** | Exclosure | Other | 2002 | 0 | . | . | . | . |
| **Treatment*Spp*Year** | Exclosure | Other | 2009 | 0 | . | . | . | . |
| **Treatment*Spp*Year** | Exclosure | Other | 2016 | 0 | . | . | . | . |
| **Treatment*Spp*Year** | Exclosure | Other | 2019 | 0 | . | . | . | . |
| **Treatment*Spp*Year** | Exclosure | etbc | 1999 | 0 | . | . | . | . |
| **Treatment*Spp*Year** | Exclosure | etbc | 2002 | 0 | . | . | . | . |
| **Treatment*Spp*Year** | Exclosure | etbc | 2009 | 0 | . | . | . | . |
| **Treatment*Spp*Year** | Exclosure | etbc | 2016 | 0 | . | . | . | . |
| **Treatment*Spp*Year** | Exclosure | etbc | 2019 | 0 | . | . | . | . |
| **Treatment*Spp*Year** | Exclosure | gten | 1999 | 0 | . | . | . | . |
| **Treatment*Spp*Year** | Exclosure | gten | 2002 | 0 | . | . | . | . |
| **Treatment*Spp*Year** | Exclosure | gten | 2009 | 0 | . | . | . | . |
| **Treatment*Spp*Year** | Exclosure | gten | 2016 | 0 | . | . | . | . |
| **Treatment*Spp*Year** | Exclosure | gten | 2019 | 0 | . | . | . | . |
| **Treatment*Spp*Year** | Exclosure | mel | 1999 | 0 | . | . | . | . |
| **Treatment*Spp*Year** | Exclosure | mel | 2002 | 0 | . | . | . | . |
| **Treatment*Spp*Year** | Exclosure | mel | 2009 | 0 | . | . | . | . |
| **Treatment*Spp*Year** | Exclosure | mel | 2016 | 0 | . | . | . | . |
| **Treatment*Spp*Year** | Exclosure | mel | 2019 | 0 | . | . | . | . |

| **Type III Tests of Fixed Effects** | | | | |
| --- | --- | --- | --- | --- |
| **Effect** | **Num DF** | **Den DF** | **F Value** | **Pr > F** |
| **Year** | 4 | 13.04 | 0.00 | 1.0000 |
| **Treatment** | 1 | 16.06 | 0.00 | 1.0000 |
| **Treatment*Year** | 4 | 13.04 | 0.00 | 1.0000 |
| **Spp** | 3 | 16.06 | 1.38 | 0.2841 |
| **Spp*Year** | 12 | 18.91 | 5.38 | 0.0006 |
| **Treatment*Spp** | 3 | 16.06 | 0.07 | 0.9732 |
| **Treatment*Spp*Year** | 12 | 18.91 | 2.92 | 0.0183 |

| **Treatment*Spp*Year Least Squares Means** | | | | | | | |
| --- | --- | --- | --- | --- | --- | --- | --- |
| **Treatment** | **Spp** | **Year** | **Estimate** | **Standard Error** | **DF** | **t Value** | **Pr > \|t\|** |
| Control | Other | 1999 | 32.1035 | 10.1948 | 15.4 | 3.15 | 0.0064 |
| Control | Other | 2002 | 31.7557 | 9.2888 | 15.77 | 3.42 | 0.0036 |
| Control | Other | 2009 | 33.6510 | 10.3568 | 16.3 | 3.25 | 0.0049 |
| Control | Other | 2016 | 35.3185 | 10.8219 | 16.39 | 3.26 | 0.0048 |
| Control | Other | 2019 | 36.2596 | 11.0989 | 16.37 | 3.27 | 0.0047 |
| Control | etbc | 1999 | 25.8867 | 10.1948 | 15.4 | 2.54 | 0.0223 |
| Control | etbc | 2002 | 26.5681 | 9.2888 | 15.77 | 2.86 | 0.0115 |
| Control | etbc | 2009 | 28.7304 | 10.3568 | 16.3 | 2.77 | 0.0134 |
| Control | etbc | 2016 | 30.7494 | 10.8219 | 16.39 | 2.84 | 0.0116 |
| Control | etbc | 2019 | 32.2364 | 11.0989 | 16.37 | 2.90 | 0.0102 |
| Control | gten | 1999 | 12.2601 | 10.1948 | 15.4 | 1.20 | 0.2473 |
| Control | gten | 2002 | 12.1727 | 9.2888 | 15.77 | 1.31 | 0.2088 |
| Control | gten | 2009 | 9.6649 | 10.3568 | 16.3 | 0.93 | 0.3643 |
| Control | gten | 2016 | 10.7469 | 10.8219 | 16.39 | 0.99 | 0.3351 |
| Control | gten | 2019 | 10.3617 | 11.0989 | 16.37 | 0.93 | 0.3641 |
| Control | mel | 1999 | 29.7497 | 10.1948 | 15.4 | 2.92 | 0.0104 |
| Control | mel | 2002 | 29.5035 | 9.2888 | 15.77 | 3.18 | 0.0059 |
| Control | mel | 2009 | 27.9537 | 10.3568 | 16.3 | 2.70 | 0.0156 |
| Control | mel | 2016 | 23.1853 | 10.8219 | 16.39 | 2.14 | 0.0475 |
| Control | mel | 2019 | 21.1424 | 11.0989 | 16.37 | 1.90 | 0.0745 |
| Exclosure | Other | 1999 | 37.3947 | 10.1948 | 15.4 | 3.67 | 0.0022 |
| Exclosure | Other | 2002 | 37.9275 | 9.2888 | 15.77 | 4.08 | 0.0009 |
| Exclosure | Other | 2009 | 29.1690 | 10.3568 | 16.3 | 2.82 | 0.0122 |
| Exclosure | Other | 2016 | 30.9492 | 10.8219 | 16.39 | 2.86 | 0.0111 |
| Exclosure | Other | 2019 | 33.3010 | 11.0989 | 16.37 | 3.00 | 0.0083 |
| Exclosure | etbc | 1999 | 22.9311 | 10.1948 | 15.4 | 2.25 | 0.0395 |
| Exclosure | etbc | 2002 | 21.5139 | 9.2888 | 15.77 | 2.32 | 0.0344 |
| Exclosure | etbc | 2009 | 25.6626 | 10.3568 | 16.3 | 2.48 | 0.0245 |
| Exclosure | etbc | 2016 | 25.4829 | 10.8219 | 16.39 | 2.35 | 0.0313 |
| Exclosure | etbc | 2019 | 25.8516 | 11.0989 | 16.37 | 2.33 | 0.0330 |
| Exclosure | gten | 1999 | 14.7164 | 10.1948 | 15.4 | 1.44 | 0.1689 |
| Exclosure | gten | 2002 | 16.4634 | 9.2888 | 15.77 | 1.77 | 0.0956 |
| Exclosure | gten | 2009 | 16.5529 | 10.3568 | 16.3 | 1.60 | 0.1292 |
| Exclosure | gten | 2016 | 16.5994 | 10.8219 | 16.39 | 1.53 | 0.1441 |
| Exclosure | gten | 2019 | 15.6936 | 11.0989 | 16.37 | 1.41 | 0.1761 |
| Exclosure | mel | 1999 | 24.9578 | 10.1948 | 15.4 | 2.45 | 0.0268 |
| Exclosure | mel | 2002 | 24.0952 | 9.2888 | 15.77 | 2.59 | 0.0197 |
| Exclosure | mel | 2009 | 28.6155 | 10.3568 | 16.3 | 2.76 | 0.0137 |
| Exclosure | mel | 2016 | 26.9685 | 10.8219 | 16.39 | 2.49 | 0.0238 |
| Exclosure | mel | 2019 | 25.1538 | 11.0989 | 16.37 | 2.27 | 0.0373 |

| **Tests of Effect Slices for Treatment*Spp*Year Sliced By Spp*Year** | | | | | |
| --- | --- | --- | --- | --- | --- |
| **Spp** | **Year** | **Num DF** | **Den DF** | **F Value** | **Pr > F** |
| Other | 1999 | 1 | 15.4 | 0.13 | 0.7186 |
| Other | 2002 | 1 | 15.77 | 0.22 | 0.6449 |
| Other | 2009 | 1 | 16.3 | 0.09 | 0.7635 |
| Other | 2016 | 1 | 16.39 | 0.08 | 0.7788 |
| Other | 2019 | 1 | 16.37 | 0.04 | 0.8528 |
| etbc | 1999 | 1 | 15.4 | 0.04 | 0.8403 |
| etbc | 2002 | 1 | 15.77 | 0.15 | 0.7056 |
| etbc | 2009 | 1 | 16.3 | 0.04 | 0.8367 |
| etbc | 2016 | 1 | 16.39 | 0.12 | 0.7351 |
| etbc | 2019 | 1 | 16.37 | 0.17 | 0.6894 |
| gten | 1999 | 1 | 15.4 | 0.03 | 0.8669 |
| gten | 2002 | 1 | 15.77 | 0.11 | 0.7482 |
| gten | 2009 | 1 | 16.3 | 0.22 | 0.6444 |
| gten | 2016 | 1 | 16.39 | 0.15 | 0.7071 |
| gten | 2019 | 1 | 16.37 | 0.12 | 0.7384 |
| mel | 1999 | 1 | 15.4 | 0.11 | 0.7441 |
| mel | 2002 | 1 | 15.77 | 0.17 | 0.6861 |
| mel | 2009 | 1 | 16.3 | 0.00 | 0.9645 |
| mel | 2016 | 1 | 16.39 | 0.06 | 0.8078 |
| mel | 2019 | 1 | 16.37 | 0.07 | 0.8015 |

**(ix) Total canopy area**

| **Model Information** | |
| --- | --- |
| **Data Set** | SASUSER.MPALAEXCL_TREECOV_9919_BY4CATS |
| **Response Variable** | SqrtCanopy |
| **Response Distribution** | Gaussian |
| **Link Function** | Identity |
| **Variance Function** | Default |
| **Variance Matrix** | Not blocked |
| **Estimation Technique** | Restricted Maximum Likelihood |
| **Degrees of Freedom Method** | Kenward-Roger |
| **Fixed Effects SE Adjustment** | Kenward-Roger |

| **Class Level Information** | | |
| --- | --- | --- |
| **Class** | **Levels** | **Values** |
| **Treatment** | 2 | Control Exclosure |
| **Block** | 3 | Baboon Kopi Mukenya |
| **Subject** | 6 | 1 2 3 4 5 6 |
| **Spp** | 4 | Other etbc gten mel |
| **Year** | 5 | 1999 2002 2009 2016 2019 |

| **Number of Observations Read** | 120 |
| --- | --- |
| **Number of Observations Used** | 120 |

| **Dimensions** | |
| --- | --- |
| **G-side Cov. Parameters** | 3 |
| **R-side Cov. Parameters** | 1 |
| **Columns in X** | 90 |
| **Columns in Z** | 123 |
| **Subjects (Blocks in V)** | 1 |
| **Max Obs per Subject** | 120 |

| **Optimization Information** | |
| --- | --- |
| **Optimization Technique** | Dual Quasi-Newton |
| **Parameters in Optimization** | 3 |
| **Lower Boundaries** | 2 |
| **Upper Boundaries** | 0 |
| **Fixed Effects** | Profiled |
| **Residual Variance** | Profiled |
| **Starting From** | Data |

| **Iteration History** | | | | | |
| --- | --- | --- | --- | --- | --- |
| **Iteration** | **Restarts** | **Evaluations** | **Objective Function** | **Change** | **Max Gradient** |
| **0** | **0** | 4 | 594.27510361 | . | 0.02372 |
| **1** | **0** | 4 | 594.25261586 | 0.02248775 | 0.001284 |
| **2** | **0** | 2 | 594.25254359 | 0.00007226 | 0.000089 |
| **3** | **0** | 2 | 594.25254325 | 0.00000035 | 3.576E-7 |

| Convergence criterion (GCONV=1E-8) satisfied. |
| --- |

| **Estimated G matrix is not positive definite.** |
| --- |

| **Fit Statistics** | |
| --- | --- |
| **-2 Res Log Likelihood** | 594.25 |
| **AIC (smaller is better)** | 600.25 |
| **AICC (smaller is better)** | 600.57 |
| **BIC (smaller is better)** | 597.55 |
| **CAIC (smaller is better)** | 600.55 |
| **HQIC (smaller is better)** | 594.82 |
| **Generalized Chi-Square** | 72.88 |
| **Gener. Chi-Square / DF** | 0.91 |

| **Covariance Parameter Estimates** | | | |
| --- | --- | --- | --- |
| **Cov Parm** | **Subject** | **Estimate** | **Standard Error** |
| **Block** |  | 0 | . |
| **Variance** | Spp(Subject) | 28.6192 | 5.2203 |
| **CS** | Spp(Subject) | 150.71 | 55.3808 |
| **Residual** |  | 0.9110 | . |

| **Solutions for Fixed Effects** | | | | | | | | |
| --- | --- | --- | --- | --- | --- | --- | --- | --- |
| **Effect** | **Treatment** | **Spp** | **Year** | **Estimate** | **Standard Error** | **DF** | **t Value** | **Pr > \|t\|** |
| **Intercept** |  |  |  | 59.6270 | 7.7511 | 21.07 | 7.69 | <.0001 |
| **Year** |  |  | 1999 | -5.1534 | 4.4370 | 64 | -1.16 | 0.2498 |
| **Year** |  |  | 2002 | 0.7325 | 4.4370 | 64 | 0.17 | 0.8694 |
| **Year** |  |  | 2009 | 19.9779 | 4.4370 | 64 | 4.50 | <.0001 |
| **Year** |  |  | 2016 | 28.7379 | 4.4370 | 64 | 6.48 | <.0001 |
| **Year** |  |  | 2019 | 0 | . | . | . | . |
| **Treatment** | Control |  |  | -29.2359 | 10.9617 | 21.07 | -2.67 | 0.0144 |
| **Treatment** | Exclosure |  |  | 0 | . | . | . | . |
| **Treatment*Year** | Control |  | 1999 | 21.0187 | 6.2748 | 64 | 3.35 | 0.0014 |
| **Treatment*Year** | Control |  | 2002 | 13.9790 | 6.2748 | 64 | 2.23 | 0.0294 |
| **Treatment*Year** | Control |  | 2009 | -8.4056 | 6.2748 | 64 | -1.34 | 0.1851 |
| **Treatment*Year** | Control |  | 2016 | -22.1099 | 6.2748 | 64 | -3.52 | 0.0008 |
| **Treatment*Year** | Control |  | 2019 | 0 | . | . | . | . |
| **Treatment*Year** | Exclosure |  | 1999 | 0 | . | . | . | . |
| **Treatment*Year** | Exclosure |  | 2002 | 0 | . | . | . | . |
| **Treatment*Year** | Exclosure |  | 2009 | 0 | . | . | . | . |
| **Treatment*Year** | Exclosure |  | 2016 | 0 | . | . | . | . |
| **Treatment*Year** | Exclosure |  | 2019 | 0 | . | . | . | . |
| **Spp** |  | Other |  | -29.1487 | 10.9617 | 21.07 | -2.66 | 0.0147 |
| **Spp** |  | etbc |  | -4.2165 | 10.9617 | 21.07 | -0.38 | 0.7043 |
| **Spp** |  | gten |  | -43.3042 | 10.9617 | 21.07 | -3.95 | 0.0007 |
| **Spp** |  | mel |  | 0 | . | . | . | . |
| **Spp*Year** |  | Other | 1999 | 7.9367 | 6.2748 | 64 | 1.26 | 0.2105 |
| **Spp*Year** |  | Other | 2002 | 9.2944 | 6.2748 | 64 | 1.48 | 0.1435 |
| **Spp*Year** |  | Other | 2009 | 9.7540 | 6.2748 | 64 | 1.55 | 0.1250 |
| **Spp*Year** |  | Other | 2016 | -12.0945 | 6.2748 | 64 | -1.93 | 0.0584 |
| **Spp*Year** |  | Other | 2019 | 0 | . | . | . | . |
| **Spp*Year** |  | etbc | 1999 | -17.3545 | 6.2748 | 64 | -2.77 | 0.0074 |
| **Spp*Year** |  | etbc | 2002 | -18.6640 | 6.2748 | 64 | -2.97 | 0.0041 |
| **Spp*Year** |  | etbc | 2009 | -20.5746 | 6.2748 | 64 | -3.28 | 0.0017 |
| **Spp*Year** |  | etbc | 2016 | -21.0219 | 6.2748 | 64 | -3.35 | 0.0014 |
| **Spp*Year** |  | etbc | 2019 | 0 | . | . | . | . |
| **Spp*Year** |  | gten | 1999 | -0.7917 | 6.2748 | 64 | -0.13 | 0.9000 |
| **Spp*Year** |  | gten | 2002 | -1.5542 | 6.2748 | 64 | -0.25 | 0.8052 |
| **Spp*Year** |  | gten | 2009 | -4.1986 | 6.2748 | 64 | -0.67 | 0.5058 |
| **Spp*Year** |  | gten | 2016 | -9.8809 | 6.2748 | 64 | -1.57 | 0.1203 |
| **Spp*Year** |  | gten | 2019 | 0 | . | . | . | . |
| **Spp*Year** |  | mel | 1999 | 0 | . | . | . | . |
| **Spp*Year** |  | mel | 2002 | 0 | . | . | . | . |
| **Spp*Year** |  | mel | 2009 | 0 | . | . | . | . |
| **Spp*Year** |  | mel | 2016 | 0 | . | . | . | . |
| **Spp*Year** |  | mel | 2019 | 0 | . | . | . | . |
| **Treatment*Spp** | Control | Other |  | 20.4109 | 15.5021 | 21.07 | 1.32 | 0.2021 |
| **Treatment*Spp** | Control | etbc |  | 17.6990 | 15.5021 | 21.07 | 1.14 | 0.2664 |
| **Treatment*Spp** | Control | gten |  | 18.3239 | 15.5021 | 21.07 | 1.18 | 0.2504 |
| **Treatment*Spp** | Control | mel |  | 0 | . | . | . | . |
| **Treatment*Spp** | Exclosure | Other |  | 0 | . | . | . | . |
| **Treatment*Spp** | Exclosure | etbc |  | 0 | . | . | . | . |
| **Treatment*Spp** | Exclosure | gten |  | 0 | . | . | . | . |
| **Treatment*Spp** | Exclosure | mel |  | 0 | . | . | . | . |
| **Treatment*Spp*Year** | Control | Other | 1999 | -14.7902 | 8.8740 | 64 | -1.67 | 0.1005 |
| **Treatment*Spp*Year** | Control | Other | 2002 | -17.8176 | 8.8740 | 64 | -2.01 | 0.0489 |
| **Treatment*Spp*Year** | Control | Other | 2009 | -18.3806 | 8.8740 | 64 | -2.07 | 0.0424 |
| **Treatment*Spp*Year** | Control | Other | 2016 | 7.7713 | 8.8740 | 64 | 0.88 | 0.3844 |
| **Treatment*Spp*Year** | Control | Other | 2019 | 0 | . | . | . | . |
| **Treatment*Spp*Year** | Control | etbc | 1999 | -8.5691 | 8.8740 | 64 | -0.97 | 0.3379 |
| **Treatment*Spp*Year** | Control | etbc | 2002 | -4.4041 | 8.8740 | 64 | -0.50 | 0.6214 |
| **Treatment*Spp*Year** | Control | etbc | 2009 | 6.6111 | 8.8740 | 64 | 0.74 | 0.4590 |
| **Treatment*Spp*Year** | Control | etbc | 2016 | 18.1605 | 8.8740 | 64 | 2.05 | 0.0448 |
| **Treatment*Spp*Year** | Control | etbc | 2019 | 0 | . | . | . | . |
| **Treatment*Spp*Year** | Control | gten | 1999 | -10.8347 | 8.8740 | 64 | -1.22 | 0.2266 |
| **Treatment*Spp*Year** | Control | gten | 2002 | -9.8936 | 8.8740 | 64 | -1.11 | 0.2691 |
| **Treatment*Spp*Year** | Control | gten | 2009 | -4.8421 | 8.8740 | 64 | -0.55 | 0.5872 |
| **Treatment*Spp*Year** | Control | gten | 2016 | 5.5656 | 8.8740 | 64 | 0.63 | 0.5328 |
| **Treatment*Spp*Year** | Control | gten | 2019 | 0 | . | . | . | . |
| **Treatment*Spp*Year** | Control | mel | 1999 | 0 | . | . | . | . |
| **Treatment*Spp*Year** | Control | mel | 2002 | 0 | . | . | . | . |
| **Treatment*Spp*Year** | Control | mel | 2009 | 0 | . | . | . | . |
| **Treatment*Spp*Year** | Control | mel | 2016 | 0 | . | . | . | . |
| **Treatment*Spp*Year** | Control | mel | 2019 | 0 | . | . | . | . |
| **Treatment*Spp*Year** | Exclosure | Other | 1999 | 0 | . | . | . | . |
| **Treatment*Spp*Year** | Exclosure | Other | 2002 | 0 | . | . | . | . |
| **Treatment*Spp*Year** | Exclosure | Other | 2009 | 0 | . | . | . | . |
| **Treatment*Spp*Year** | Exclosure | Other | 2016 | 0 | . | . | . | . |
| **Treatment*Spp*Year** | Exclosure | Other | 2019 | 0 | . | . | . | . |
| **Treatment*Spp*Year** | Exclosure | etbc | 1999 | 0 | . | . | . | . |
| **Treatment*Spp*Year** | Exclosure | etbc | 2002 | 0 | . | . | . | . |
| **Treatment*Spp*Year** | Exclosure | etbc | 2009 | 0 | . | . | . | . |
| **Treatment*Spp*Year** | Exclosure | etbc | 2016 | 0 | . | . | . | . |
| **Treatment*Spp*Year** | Exclosure | etbc | 2019 | 0 | . | . | . | . |
| **Treatment*Spp*Year** | Exclosure | gten | 1999 | 0 | . | . | . | . |
| **Treatment*Spp*Year** | Exclosure | gten | 2002 | 0 | . | . | . | . |
| **Treatment*Spp*Year** | Exclosure | gten | 2009 | 0 | . | . | . | . |
| **Treatment*Spp*Year** | Exclosure | gten | 2016 | 0 | . | . | . | . |
| **Treatment*Spp*Year** | Exclosure | gten | 2019 | 0 | . | . | . | . |
| **Treatment*Spp*Year** | Exclosure | mel | 1999 | 0 | . | . | . | . |
| **Treatment*Spp*Year** | Exclosure | mel | 2002 | 0 | . | . | . | . |
| **Treatment*Spp*Year** | Exclosure | mel | 2009 | 0 | . | . | . | . |
| **Treatment*Spp*Year** | Exclosure | mel | 2016 | 0 | . | . | . | . |
| **Treatment*Spp*Year** | Exclosure | mel | 2019 | 0 | . | . | . | . |

| **Type III Tests of Fixed Effects** | | | | |
| --- | --- | --- | --- | --- |
| **Effect** | **Num DF** | **Den DF** | **F Value** | **Pr > F** |
| **Year** | 4 | 64 | 27.95 | <.0001 |
| **Treatment** | 1 | 16 | 10.82 | 0.0046 |
| **Treatment*Year** | 4 | 64 | 27.29 | <.0001 |
| **Spp** | 3 | 16 | 10.92 | 0.0004 |
| **Spp*Year** | 12 | 64 | 4.57 | <.0001 |
| **Treatment*Spp** | 3 | 16 | 0.68 | 0.5760 |
| **Treatment*Spp*Year** | 12 | 64 | 1.92 | 0.0479 |

| **Treatment*Spp*Year Least Squares Means** | | | | | | | |
| --- | --- | --- | --- | --- | --- | --- | --- |
| **Treatment** | **Spp** | **Year** | **Estimate** | **Standard Error** | **DF** | **t Value** | **Pr > \|t\|** |
| Control | Other | 1999 | 30.6651 | 7.7511 | 21.07 | 3.96 | 0.0007 |
| Control | Other | 2002 | 27.8416 | 7.7511 | 21.07 | 3.59 | 0.0017 |
| Control | Other | 2009 | 24.5989 | 7.7511 | 21.07 | 3.17 | 0.0046 |
| Control | Other | 2016 | 23.9580 | 7.7511 | 21.07 | 3.09 | 0.0055 |
| Control | Other | 2019 | 21.6533 | 7.7511 | 21.07 | 2.79 | 0.0109 |
| Control | etbc | 1999 | 33.8153 | 7.7511 | 21.07 | 4.36 | 0.0003 |
| Control | etbc | 2002 | 35.5170 | 7.7511 | 21.07 | 4.58 | 0.0002 |
| Control | etbc | 2009 | 41.4824 | 7.7511 | 21.07 | 5.35 | <.0001 |
| Control | etbc | 2016 | 47.6402 | 7.7511 | 21.07 | 6.15 | <.0001 |
| Control | etbc | 2019 | 43.8736 | 7.7511 | 21.07 | 5.66 | <.0001 |
| Control | gten | 1999 | 9.6497 | 7.7511 | 21.07 | 1.24 | 0.2268 |
| Control | gten | 2002 | 8.6745 | 7.7511 | 21.07 | 1.12 | 0.2757 |
| Control | gten | 2009 | 7.9424 | 7.7511 | 21.07 | 1.02 | 0.3171 |
| Control | gten | 2016 | 7.7235 | 7.7511 | 21.07 | 1.00 | 0.3303 |
| Control | gten | 2019 | 5.4108 | 7.7511 | 21.07 | 0.70 | 0.4928 |
| Control | mel | 1999 | 46.2564 | 7.7511 | 21.07 | 5.97 | <.0001 |
| Control | mel | 2002 | 45.1026 | 7.7511 | 21.07 | 5.82 | <.0001 |
| Control | mel | 2009 | 41.9634 | 7.7511 | 21.07 | 5.41 | <.0001 |
| Control | mel | 2016 | 37.0191 | 7.7511 | 21.07 | 4.78 | 0.0001 |
| Control | mel | 2019 | 30.3911 | 7.7511 | 21.07 | 3.92 | 0.0008 |
| Exclosure | Other | 1999 | 33.2616 | 7.7511 | 21.07 | 4.29 | 0.0003 |
| Exclosure | Other | 2002 | 40.5052 | 7.7511 | 21.07 | 5.23 | <.0001 |
| Exclosure | Other | 2009 | 60.2102 | 7.7511 | 21.07 | 7.77 | <.0001 |
| Exclosure | Other | 2016 | 47.1217 | 7.7511 | 21.07 | 6.08 | <.0001 |
| Exclosure | Other | 2019 | 30.4783 | 7.7511 | 21.07 | 3.93 | 0.0008 |
| Exclosure | etbc | 1999 | 32.9026 | 7.7511 | 21.07 | 4.24 | 0.0004 |
| Exclosure | etbc | 2002 | 37.4791 | 7.7511 | 21.07 | 4.84 | <.0001 |
| Exclosure | etbc | 2009 | 54.8138 | 7.7511 | 21.07 | 7.07 | <.0001 |
| Exclosure | etbc | 2016 | 63.1265 | 7.7511 | 21.07 | 8.14 | <.0001 |
| Exclosure | etbc | 2019 | 55.4105 | 7.7511 | 21.07 | 7.15 | <.0001 |
| Exclosure | gten | 1999 | 10.3778 | 7.7511 | 21.07 | 1.34 | 0.1949 |
| Exclosure | gten | 2002 | 15.5012 | 7.7511 | 21.07 | 2.00 | 0.0586 |
| Exclosure | gten | 2009 | 32.1022 | 7.7511 | 21.07 | 4.14 | 0.0005 |
| Exclosure | gten | 2016 | 35.1799 | 7.7511 | 21.07 | 4.54 | 0.0002 |
| Exclosure | gten | 2019 | 16.3229 | 7.7511 | 21.07 | 2.11 | 0.0474 |
| Exclosure | mel | 1999 | 54.4737 | 7.7511 | 21.07 | 7.03 | <.0001 |
| Exclosure | mel | 2002 | 60.3596 | 7.7511 | 21.07 | 7.79 | <.0001 |
| Exclosure | mel | 2009 | 79.6050 | 7.7511 | 21.07 | 10.27 | <.0001 |
| Exclosure | mel | 2016 | 88.3650 | 7.7511 | 21.07 | 11.40 | <.0001 |
| Exclosure | mel | 2019 | 59.6270 | 7.7511 | 21.07 | 7.69 | <.0001 |

| **Tests of Effect Slices for Treatment*Spp*Year Sliced By Spp*Year** | | | | | |
| --- | --- | --- | --- | --- | --- |
| **Spp** | **Year** | **Num DF** | **Den DF** | **F Value** | **Pr > F** |
| Other | 1999 | 1 | 21.07 | 0.06 | 0.8150 |
| Other | 2002 | 1 | 21.07 | 1.33 | 0.2609 |
| Other | 2009 | 1 | 21.07 | 10.55 | 0.0038 |
| Other | 2016 | 1 | 21.07 | 4.47 | 0.0467 |
| Other | 2019 | 1 | 21.07 | 0.65 | 0.4298 |
| etbc | 1999 | 1 | 21.07 | 0.01 | 0.9344 |
| etbc | 2002 | 1 | 21.07 | 0.03 | 0.8597 |
| etbc | 2009 | 1 | 21.07 | 1.48 | 0.2374 |
| etbc | 2016 | 1 | 21.07 | 2.00 | 0.1723 |
| etbc | 2019 | 1 | 21.07 | 1.11 | 0.3045 |
| gten | 1999 | 1 | 21.07 | 0.00 | 0.9477 |
| gten | 2002 | 1 | 21.07 | 0.39 | 0.5401 |
| gten | 2009 | 1 | 21.07 | 4.86 | 0.0388 |
| gten | 2016 | 1 | 21.07 | 6.27 | 0.0205 |
| gten | 2019 | 1 | 21.07 | 0.99 | 0.3308 |
| mel | 1999 | 1 | 21.07 | 0.56 | 0.4618 |
| mel | 2002 | 1 | 21.07 | 1.94 | 0.1785 |
| mel | 2009 | 1 | 21.07 | 11.79 | 0.0025 |
| mel | 2016 | 1 | 21.07 | 21.94 | 0.0001 |
| mel | 2019 | 1 | 21.07 | 7.11 | 0.0144 |


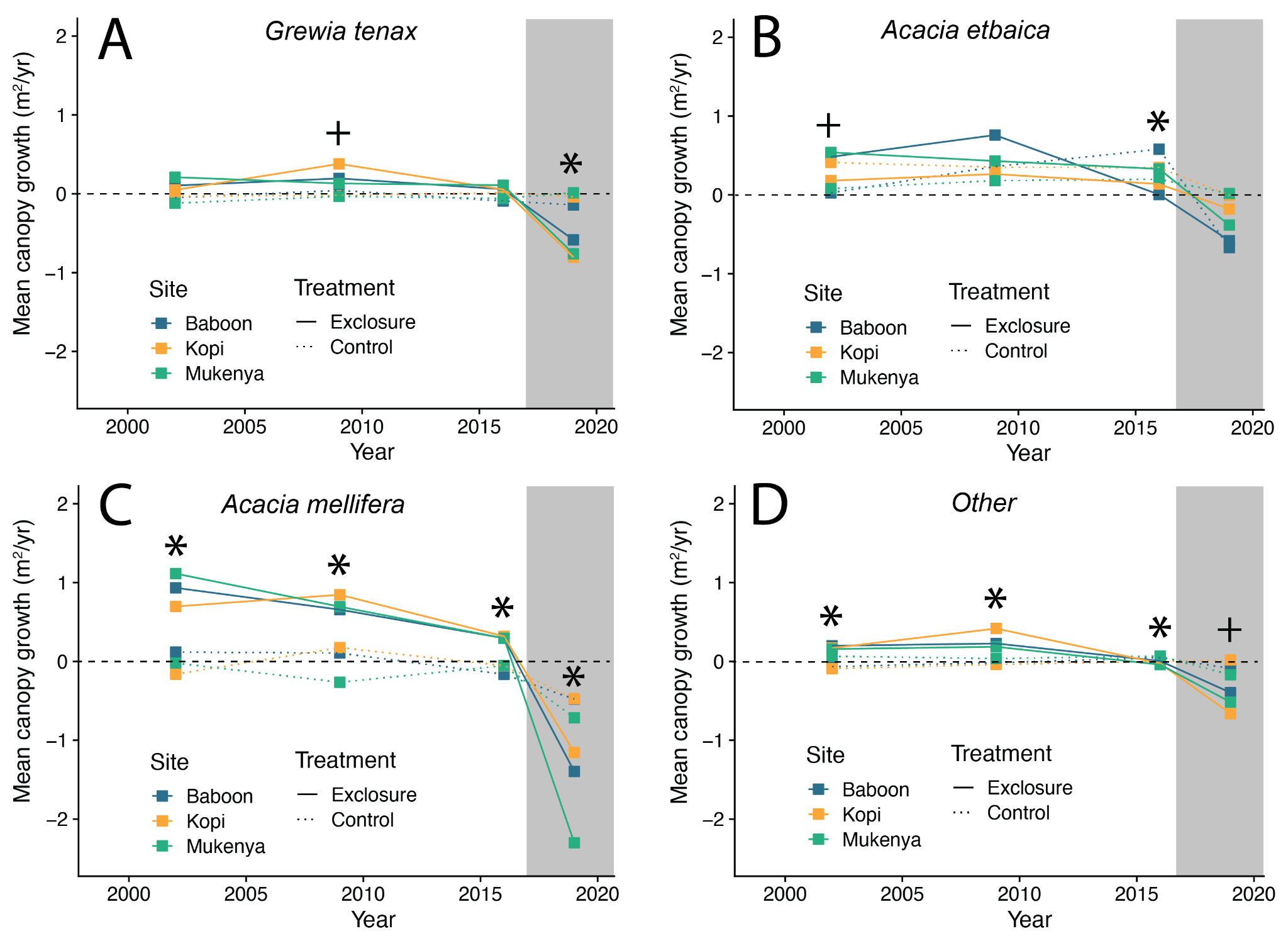


**Figure S1.** Mean canopy area growth (m^2^ yr^-1^) of (A) *Grewia tenax*, (B) *Acacia etbaica*, (C) *A. mellifera*, and (D) all other tree species. Growth rates are measured by calculating the change in canopy area between sequential survey years and dividing by the number of years between surveys; rates are reported for the end date of each intersurvey period (*e.g.*, rates displayed in 2002 represent annual growth rates between 1999 and 2002). The dashed horizontal line denotes zero average growth (*i.e.*, no change in canopy area over time). Data are averaged at the plot level (N = 3 plots/treatment). The grey box denotes the period of herbivore reintroduction, which occurred one year after the 2016 surveys. (*) denotes significant treatment differences at *P <* 0.05; (+) denotes significant treatment differences at 0.05 < *P <* 0.10. Note that the (*) in (B) denotes greater growth in control than exclosure for *A. etbaica* from 2009-2016.

**
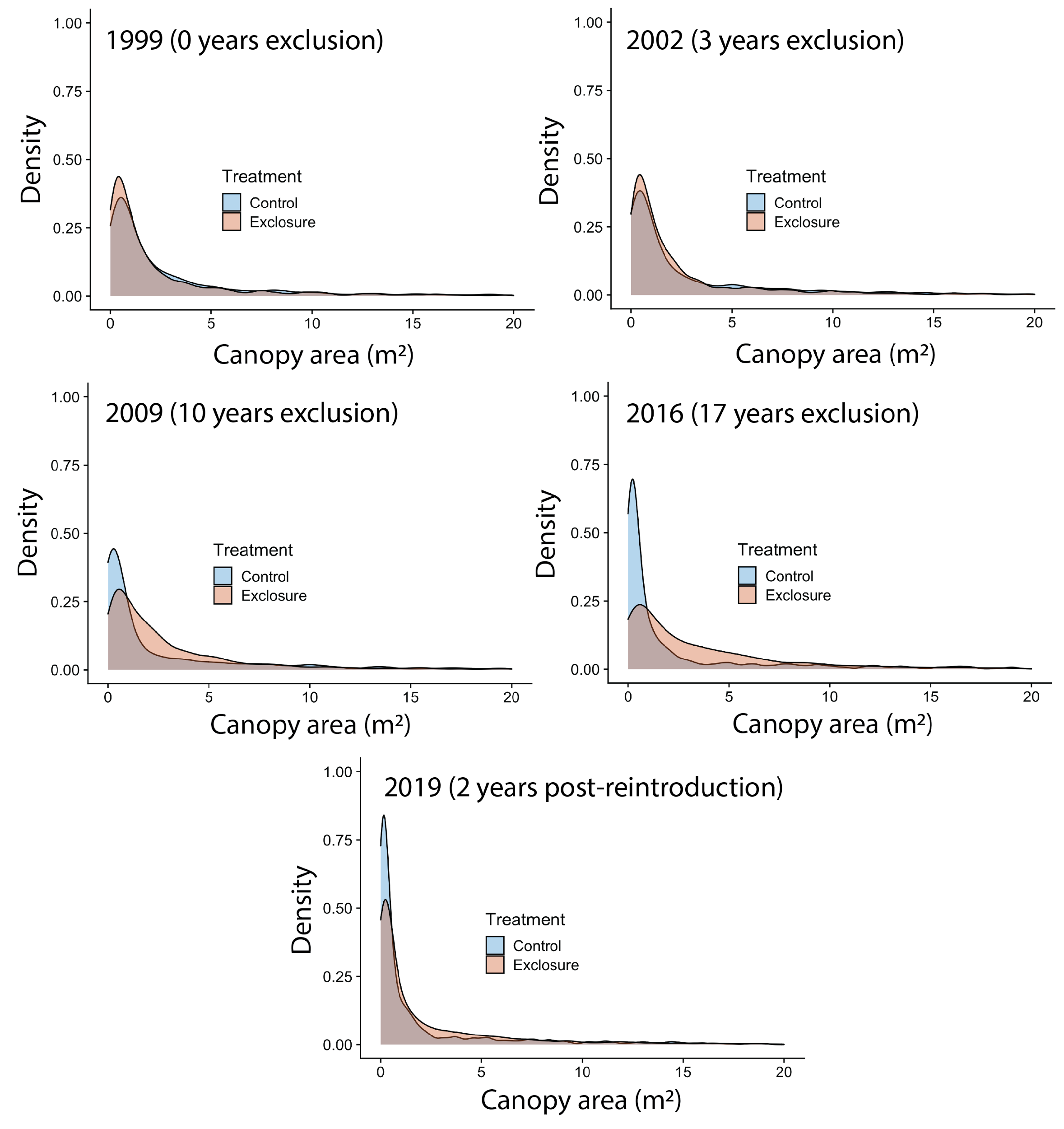
**

**Figure S2.** Density plots of individual canopy areas (m^2^) across treatments and years.


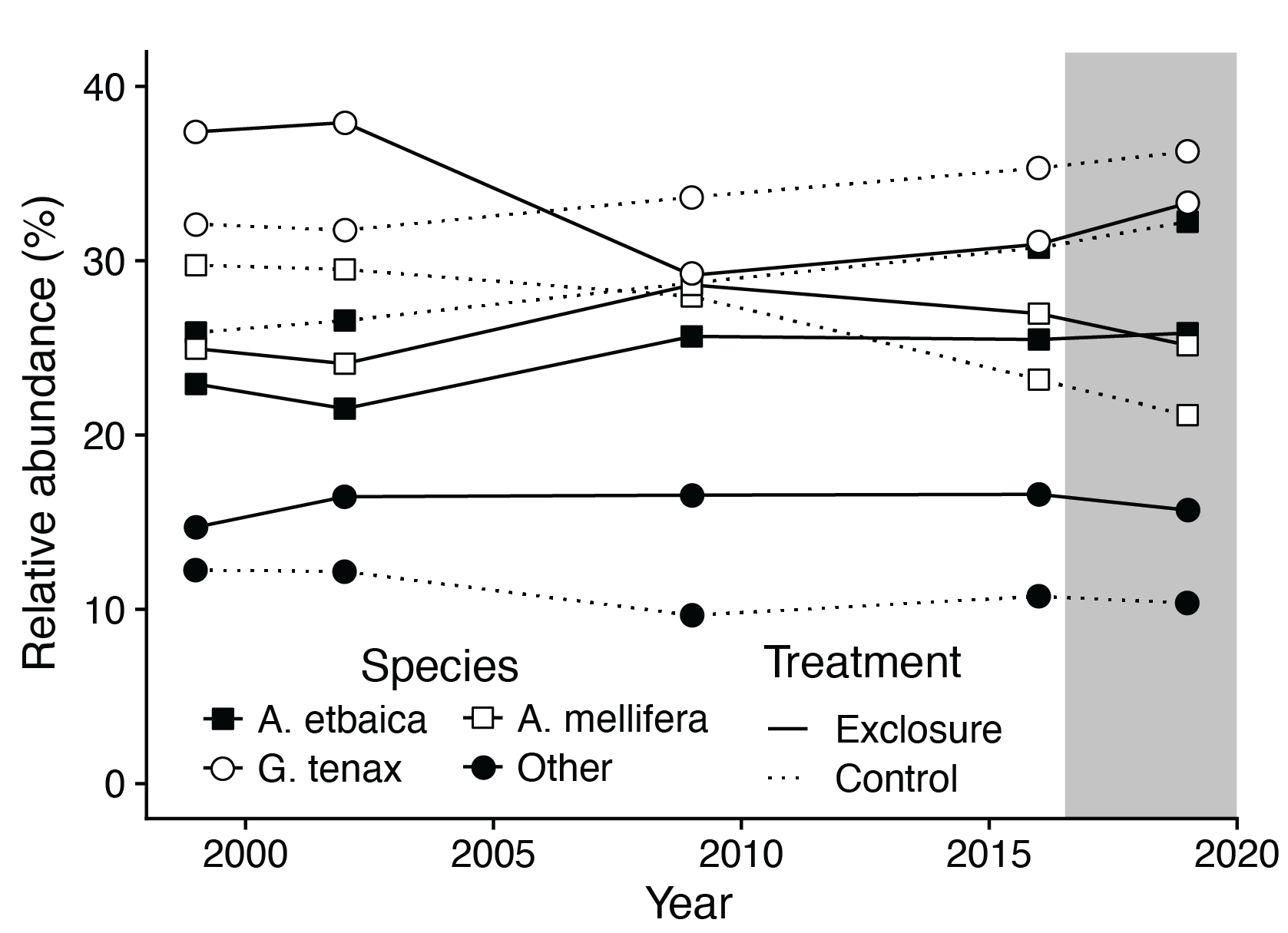


**Figure S3.** Composition of the tree community in exclosure and control plots across time. Relative abundance was calculated as the proportion (%) of all trees in each plot belonging to each of three species: *Acacia etbaica, A. mellifera, Grewia tenax*; all other species were lumped and are designated “Other” in the figure. Solid lines denote exclosure plots; dashed lines denote unfenced control plots. The 1999 survey represents pre-exclosure baseline conditions. The grey box denotes the period of herbivore reintroduction, which occurred one year after the 2016 surveys. See also Figure 3, which represents a cover-based estimate of community composition.

**References:**

Alston, J.M., Reed, C.G., Khasoha, L.M., Brown, B.R.P., Busienei, G., Carlson, N., *et al.* (2022). Ecological consequences of large herbivore exclusion in an African savanna: 12 years of data from the UHURU experiment. *Ecology*, 103, e3649.

Axelsson, P. 2000. DEM generation from laser scanner data using adaptive TIN models. International Archives of Photogrammetry and Remote Sensing 33:110–117.

Boucher, P. B., E. G. Hockridge, J. Singh, and A. B. Davies. 2023. Flying high: Sampling savanna vegetation with UAV-lidar. Methods in Ecology and Evolution 14:1668–1686.

Coverdale, T.C., Boucher, P.B., Singh, J., Palmer, T.M., Goheen, J.R., Pringle, R.M., *et al.* (2024). Herbivore regulation of savanna vegetation: Structural complexity, diversity, and the complexity–diversity relationship. *Ecol Monogr*, 94, e1624.

Goheen, J.R., Palmer, T.M., Charles, G.K., Helgen, K.M., Kinyua, S.N., Maclean, J.E., *et al.* (2013). Piecewise disassembly of a large-herbivore community across a rainfall gradient: The UHURU experiment. *PLoS One*, 8, e55192.
